# Classification and Prognostic Characteristics of Hepatocellular Carcinoma Based on Glycolysis Cholesterol Synthesis Axis

**DOI:** 10.1155/2022/2014625

**Published:** 2022-09-30

**Authors:** Weijie Deng, Peng Zhu, Huixuan Xu, Xianliang Hou, Wenbiao Chen

**Affiliations:** ^1^Clinical Skills Center, Shenzhen Pingshan District People's Hospital, Pingshan General Hospital, Southern Medical University, Shenzhen 518000, China; ^2^Central Laboratory, Shenzhen Pingshan District People's Hospital, Pingshan General Hospital, Southern Medical University, Shenzhen 518000, China; ^3^Department of Clinical Medical Research Center, The Second Clinical Medical College of Jinan University, The First Affiliated Hospital Southern University of Science and Technology, Shenzhen People's Hospital, Shenzhen 518000, China; ^4^Central Molecular Laboratory, People's Hospital of Longhua, The Affiliated Hospital of Southern Medical University, Shenzhen 518000, China; ^5^Department of Respiratory Medicine, People's Hospital of Longhua, The Affiliated Hospital of Southern Medical University, Shenzhen 518000, China

## Abstract

**Background:**

Liver hepatocellular carcinoma (LIHC) is among the most frequent causes of cancer-related death across the world with a considerably poor prognosis. The current study targeted providing a new type of LIHC from the perspective of the glycolysis/cholesterol synthesis axis, predicting its prognostic characteristics, and exploring the potential role and mechanism of the glycolysis/cholesterol synthesis axis in the occurrence and development of LIHC.

**Methods:**

Based on the two expression profile data and clinical information of LIHC in The Cancer Genome Atlas (TCGA) database and hepatocellular carcinoma database (HCCDB), as well as glycolysis/cholesterol-related genes from the Molecular Signatures Database (MSigDB), unsupervised consistent clustering method was used to identify molecular subtypes. In addition, the differential genes were identified by limma package, and then the gene set was enriched, analyzed, and annotated by WebGestaltR package. At the same time, the immune infiltration analysis of tumor samples was carried out using the ESTIMATE to evaluate the tumor immune score of the samples. Finally, the differences in clinical characteristics among molecular subtypes were measured using univariate and multivariate Cox analyses.

**Results:**

According to the median standardized expression levels of glycolysis/cholesterol production genes, samples were divided into four groups (molecular subtypes): Quiescent group, Glycolysis group, Cholesterol group, and Mixed group. Significant prognostic differences were observed among the four groups. In both TCGA and HCCDB18 datasets, the prognosis of subtype Mixed was the worst, while Quiescent had a good prognosis. Cell cycle and oncogenic pathways were significantly enriched in the Mixed group. In addition, glycolysis and cholesterol production gene expressions were related to the prognostic LIHC subtype classification genes' expression levels.

**Conclusion:**

Metabolic classification regarding glycolysis and cholesterol production pathways provided new insights into the biological aspects of LIHC molecular subtypes and might help to develop personalized therapies for unique tumor metabolic profiles.

## 1. Introduction

Liver hepatocellular carcinoma (LIHC) is the top frequent primary cancer type of the liver around the world. Its incidence rate has shown to be rising and closely related to advanced liver disease [1–3]. This malignancy is the main cause of death in patients with liver cirrhosis. At the same time, liver cirrhosis is also an important indicator for monitoring and screening the occurrence of liver cancer [4–6]. Despite considerable progress in surgical therapeutic approaches and medical measures, liver cancer is still one of the most common tumor-related causes of death around the world. Most LIHC patients are diagnosed at advanced stages and have no opportunity for surgical resection [1], and most patients after surgery are also very poor [7–9]. Patients with similar tumor stages or pathological structures may have significantly different prognoses due to individual differences [10, 11]. Therefore, it is critical to explore new molecular subtypes to predict the prognostic characteristics of different LIHC.

Metabolic reprogramming in cancerous cells driven by oncogenes enables them to survive and proliferate in the complex microenvironment of tumors [12]. Pan-cancer analyses of global metabolic pathways show that tumor metabolism is heterogeneous in terms of survival, somatic driver gene mutations, and tumor subtypes [13]. Although, hepatocytes have never been fully determined to be feasible to be divided into clinically relevant groups according to their heterogeneity in metabolic pathways. Oncogenic CTNNB1 and TP53 mutations resulting in function loss are known to be inducing glycolysis pathways in cancer, which contribute to the progression of tumors and developing resistance to chemotherapy in tumors [14, 15]. The role of glycolytic pathways in tumor progression can be weakened by inhibiting the pyruvate to lactic acid conversion but transferring this metabolite to mitochondria through mitochondrial pyruvate complex (MPC) composed of two complexes: MPC1 and MPC2 [16–18]. Decreased MPC function is associated with a poorer prognosis in several types of cancer [18]. Pyruvate is a metabolite mediating the tricarboxylic acid cycle, which provides citrate (a precursor of adipogenesis) which involve in the biological synthesis of cholesterol and free fatty acids [12, 19]. The Mevalonate pathway is another axis that essentially contributes to *de novo* synthesis of cholesterol and can be induced by oncogenes contributing to tumor growth. These findings support the benefit of using pathway inhibitors such as statins for treating cancer [20]. Although, research studying the relationship between statins and cancer survival rate or risk has represented controversial outcomes [21–24]. The heterogeneity in responses to statin might be related to different molecular characteristics of tumors [20, 22, 25]. The relationship between the MPC1 and MPC2 expression and tumor prognosis increases the cross-tumor difference in pyruvate flow and the possible involvement of glycolysis/cholesterol synthesis balance in the regulation of tumor invasiveness [18].

The main objective of the present study was to identify the molecular subtypes of LIHC with prognostic properties. For this purpose, LIHC patients were divided into subtypes according to the expression of glycolysis/cholesterol synthesis-related genes. Different subtypes were then screened for differences in their survival rate and other clinical features of patients with different molecular subtypes, and the molecular processes underlying carcinogenesis were identified in each subtype. The present findings have depicted a LIHC classification scheme that is conveniently applicable in clinics and can be used as a guide for developing targeted therapy for LIHC.

## 2. Methods

### 2.1. Data Download and Preprocessing

The RNA sequencing (RNA-seq) expression data, single nucleotide variation/insertions and deletions (SNV/InDel) data, copy number variation (CNV) data, and clinical follow-up information data (April 30, 2020) of the LIHC dataset (TCGA-LIHC, hereinafter referred to as TCGA) were obtained from The Cancer Genome Atlas (TCGA) database (http://cancergenome.nih.gov/abouttcga). Then, the expression profile data of the Primary Solid Tumor (TP) and Solid Tissue Normal (NT) samples were retained, and the Ensemble ID was converted into the gene symbol. In the case of multiple gene symbols, the mid-value was taken. Finally, the expression spectrum was converted from FPKM (fragments per kilobase million) format to TPM (transcripts per million). There were 421 samples in the preprocessed TCGA dataset, including 371 tumor samples and 50 normal samples.

HCCDB18dataset was downloaded in the hepatocellular carcinoma database (HCCDB) (http://lifeome.net/database/hccdb/home.html), including RNA-seq data, SNV/InDel data, and clinical follow-up information data (April 30, 2020). Subsequently, the samples lacking clinical follow-up information, survival time, status state, and expression profile data were removed. After pretreatment, there were 380 samples in the HCCDB18 dataset, including 203 tumor samples and 177 normal samples.

### 2.2. Source and Treatment of Glycolysis and Cholesterol-Related Genes

Glycolysis and cholesterol-related genes were from REACTOME_GLYCOLYSIS (29 genes) and REACTOME_CHOLESTEROL_BIOSYNTHESIS (24 genes) in c2.cp.reactome.v6.2.symbols.gmt file in the Molecular Signatures Database (MSigDB, https://www.gsea-msigdb.org/gsea/msigdb/) [26], with 53 genes in total. The expression profile data obtained from TCGA was filtered. The TCGA expression profile data were filtered. The filtering standard was to remove the genes whose expression was less than 1 and less than 50%. After filtering, only 44 glycolysis/cholesterol-related genes were retained, including 24 glycolysis-related genes and 20 cholesterol synthesis-related genes (Supplementary Table S1).

### 2.3. Consistent Clustering

The glycolysis/cholesterol synthesis-related genes were clustered by ConsensusClusterPlus package [27] (V1.48.0; parameters: reps = 100, pItem = 0.8, pFeature = 1, distance = “pearson”), using D2 clustering algorithm and Euclidean distance. Then, 500 bootstraps were carried out. Each bootstrap process included 80% of the training set patients. The cluster number (*k*) was set as 2–10. Then, the consistency cumulative distribution function (CDF) and the area under the CDF curve of each *k* value were calculated to identify the optimal classification based on the Elbow method and consensus matrix.

### 2.4. Gene Set Enrichment Analysis (GSEA) and Annotation of Differentially Expressed Genes

The difference analysis between subtypes was carried out by limma package [28], and the different genes were screened by |log2 (Fold Change)| > 1 and false discovery rate (FDR) < 0.05. Differentially expressed genes (DEGs) among subtypes were enriched and analyzed by Gene Ontology (GO) and Kyoto Encyclopedia of Genes and Genomes (KEGG) through WebGestaltRpackage (v0.4.2) [29], and the gene set was selected to be c2.cp.kegg.v7.0.symbols.gmt, which contained the KEGG pathway. GSEA input file contained expression profile data and sample labels labeled with molecular subtypes. The sample label marked the samples under either Mixed or Quiescent groups. The thresholds of enrichment pathways were *P* < 0.05 and FDR < 0.25. GO function enrichment analysis was performed on MPC1/2 positive and negative genes through the R software package WebGestaltR (the threshold was set to *P* < 0.05).

### 2.5. Analysis of the Immune Microenvironment of Tumors

The ESTIMATE (Estimation of STromal and Immune cells in MAlignant Tumor tissues using Expression data) method [30]was applied to measure the tumor immune microenvironment scores of the samples and then their differential distribution among different subtypes was compared. Based on the expression data, ESTIMATE provided researchers with a score for tumor purity, as well as the levels of stromal cells and immune cell infiltration in the tumor tissue.

### 2.6. Univariate and Multivariate Cox Analysis

R software package glmnet [31]was applied for establishing the Lasso Cox regression model. According to the constructed model, hazard ratio (HR), 95% confidence interval (CI) of HR, and P-value of clinical features and molecular subtypes were analyzed using univariate and multivariate Cox regression in the survival R package. Log-rank test was conducted to test the difference between the variables (clinical features and molecular subtypes) and overall survival. The variables with *P* < 0.05 and 95%CI of HR > 1 were considered independent risk factors.

### 2.7. Statistical Analysis

A Chi-square test was applied to explore the clinicopathological differences among the four subtypes and ANOVA was applied for the identification of the expression levels in each. The *t*-test was applied to study the difference between every two groups. Pearson correlation coefficient was used for correlation analysis. All statistical analyses were done using R software (v 4.0.2). *P* < 0.05 was considered statistically significant.

## 3. Results

### 3.1. Identification of Molecular Subtypes

**Figure 1:**
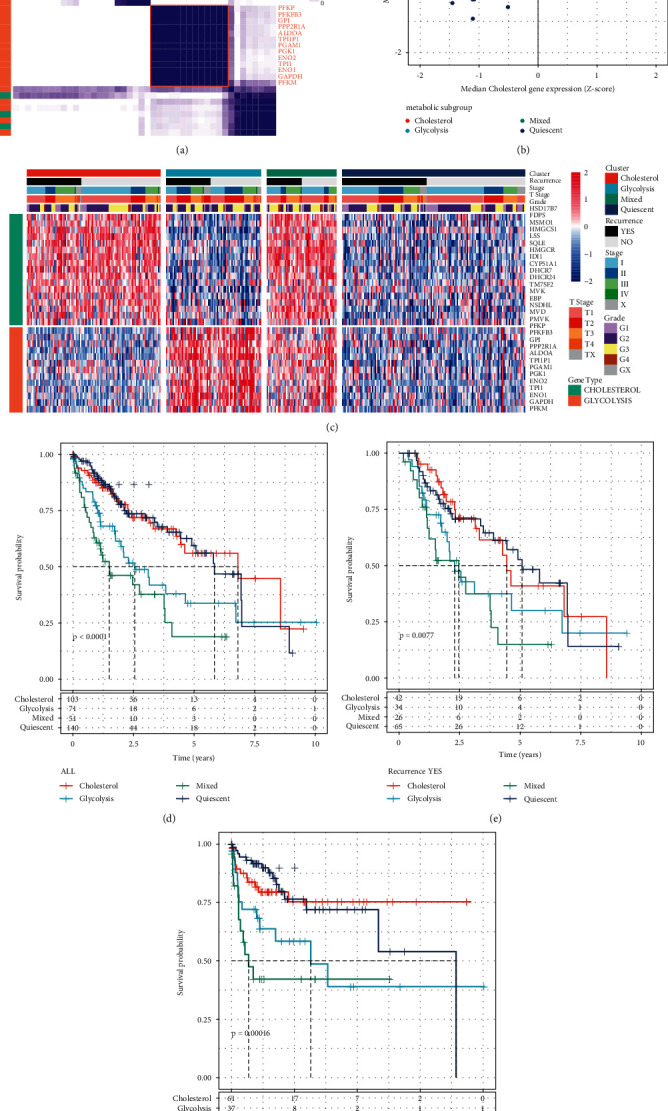
Identification of TCGA groups of liver cancer. (a) Consistent clustering of glycolysis/cholesterol synthesis-related genes. (b) Classification of samples based on glycolysis/cholesterol synthesis-related genes expression levels. (c) Cluster heatmap of 30 related genes. (d) Prognostic survival curve of molecular subtypes of all samples of liver cancer. (e) Prognostic survival curve of molecular subtypes of recurrent liver cancer samples. (f) Prognostic survival curve of molecular subtypes of non-recurrent liver cancer samples.

According to the data of TCGA, the expression level of 44 glycolytic/cholesterol synthesis-related genes was obtained and consistent clustering was performed. At *k* = 4, glycolytic genes and cholesterol genes could aggregate together, respectively (Figure 1(a)). Z-scores were calculated using median expression levels of co-expressed glycolytic/cholesterol synthesis-related genes and were then used to subtype grouping of 371 tumor samples in the TCGA dataset. Samples with GLYCOLYSIS ≤ 0 and CHOLESTEROL ≤ 0 were defined as the Quiescent group; samples with GLYCOLYSIS > 0 and CHOLESTEROL ≤ 0 were defined as the Glycolysis group; samples with GLYCOLYSIS ≤ 0 and CHOLESTEROL > 0 were defined as Cholesterol group and; samples with GLYCOLYSIS ≥ 0 and CHOLESTEROL ≥ 0 were defined as Mixed group (Figure 1(b)).

The glycolytic/cholesterol synthesis-related gene expression levels showed significant differences in the four subtypes (Figure 1(c)). Furthermore, prognostic relationship analysis between every two groups showed significant differences among all subtypes. In all datasets, the Mixed and Quiescent groups had the poorest and best prognoses, respectively (Figures 1(d)–1(f), log-rank*P* < 0.01).

### 3.2. Relationship between Molecular Mutation among Molecular Subtypes and CNV

**Figure 2:**
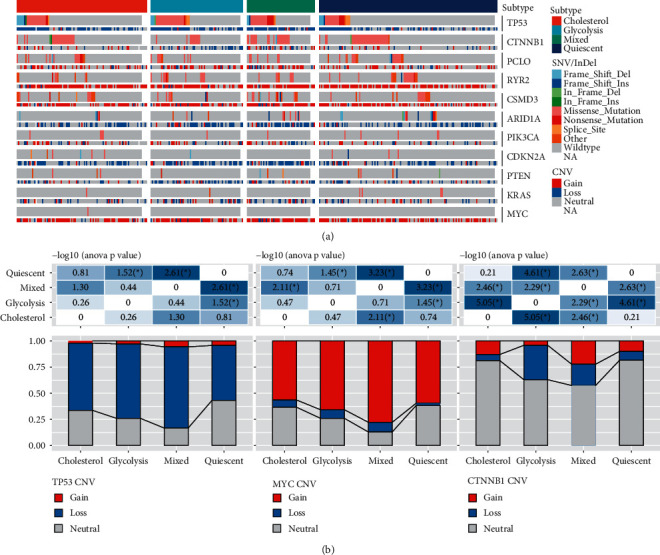
Mutation features and CNV difference among four molecular subtypes in TCGA dataset. (a) Mutation distribution among molecular subtypes. (b) Comparison of CNV differences among molecular subtype of genes TP53, MYC, and CTNNB1. ^*∗*^*P* < 0.05.

Molecular events, including oncogenic mutations such as MYC amplification and TP53 mutation, could conduct metabolic reprogramming of cancers, including LIHC [32, 33]. For identifying the difference in carcinogenic events among various molecular subtypes, genes with frequent mutations in LIHC between SNV/InDel and CNV were studied (Figure 2). TP53 and CTNNB1 mutations showed mutually exclusive behavior. The mutation frequency of each gene showed no significant difference between each subtype pair. However, significant differences were observed between the Mixed and Quiescent groups in the TP53 deletion samples. The deletion ratio was higher in the Mixed group compared to the Quiescent group. In addition, the CNV changes of MYC and CTNNB1 in the Mixed group also differed from those in the Quiescent group. The gain proportion of MYC and CTNNB1 was also significantly higher in the mixed group compared to the Quiescent group.

### 3.3. The MPC Complex as a Potential Regulatory Factor for Glycolysis/Cholesterol Synthesis Axis of the Tumor

**Figure 3:**
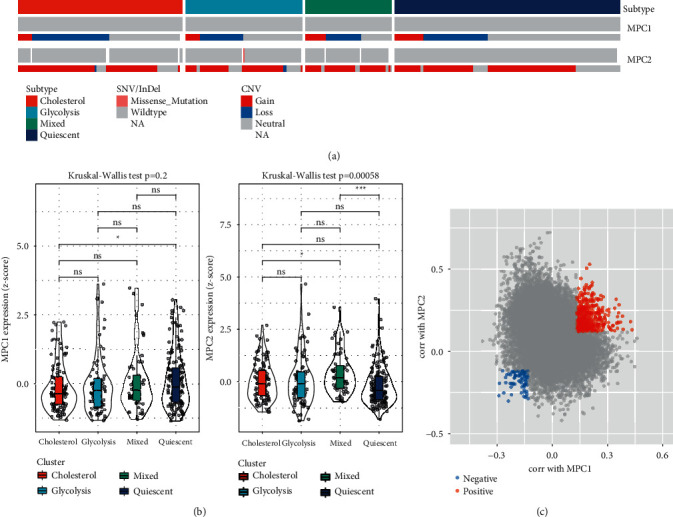
Analysis of MPC complex among different subtypes in TCGA dataset. (a) Mutations and CNV distribution of MPC1 and 2 in different molecular subtypes. (b) Comparison of MPC1/2 expression among different molecular subtypes. (c) Scatter plot of genes associated with MPC1/2. Kruskal-Wallis test was performed among four groups. ns, no significance. ^*∗*^*P* < 0.05, ^*∗∗∗*^*P* < 0.001.

MPC complex regulated mitochondrial pyruvate flow inhibited the MPC1 and MPC2 expression in cancer cells and promoted glycolysis and lactate synthesis in tumor samples [34]. For studying the relationship of MPC1 and MPC2 complexes with glycolysis and cholesterol synthesis phenotypes, their mutation frequency and expression levels were compared in the molecular subtypes. It was found that the contradictory relationship of CNV in each gene, in which CNV affected MPC1 mainly by deletion, while most of CNV affecting MPC2 was amplification (Figure 3(a)). There was no significant difference in MPC1 among molecular subtypes, but their MPC2 expression levels were different. Also, the expression of MPC2 complex was significantly higher in the mixed group compared to the Quiescent and Cholesterol groups (Figure 3(b)). In order to find the cellular pathway relating to the MPC1/2 expression, a comprehensive correlation analysis was conducted between all other tested genes and theirs. A total of 519 and 83 genes showed positive and negative correlation with MPC1 and MPC2 complexes, respectively (Spearman correlation, BH correction *P* < 0.05) (Figure 3(c)). Further analyses proved that positively related genes to MPC1 and MPC2 were associated with positive regulation of steroid and lipid metabolisms as well as an extracellular matrix organization (Supplementary Figures S1(a)–S1(c)). Whereas negatively related genes to MPC1/2 showed a positive regulatory effect on the peptide hormone secretion (Supplementary Figures S1(d) and S1(e)).

### 3.4. Validation of the Subtyping in the HCCDB18 Dataset

**Figure 4:**
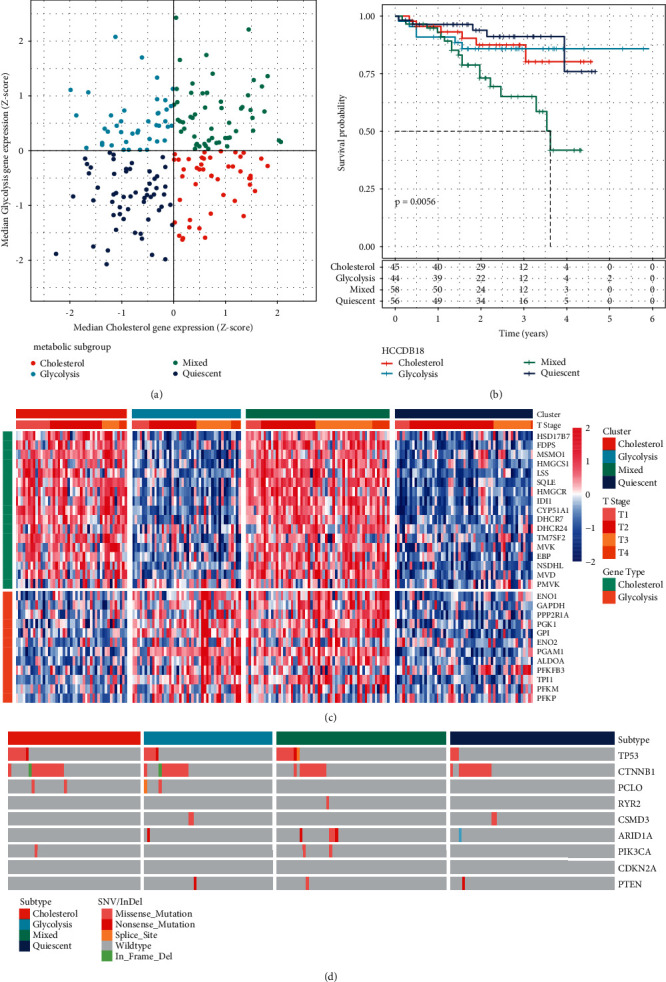
Subtype validation in HCCDB18 dataset. (a) Classification of samples according to glycolysis/cholesterol synthesis-related gene expression levels. (b) Prognostic survival curve of molecular subtypes of HCCDB18 liver cancer in all samples. (c) Cluster heatmap of 29 related genes. (d) Mutation patterns of the top nine mutated genes in four molecular subtypes.

To verify the analysis results of molecular subtypes in the earlier TCGA dataset, the expression profiles of co-expressed genes related to glycolysis and cholesterol were extracted from the HCCDB18 dataset, respectively. Among them, the TPI1P1 gene related to glycolysis does not exist in the HCCDB18 dataset; then, glycolysis used the remaining 12 genes, and cholesterol used 17 genes. The median expression levels of co-expressed glycolytic and cholesterol-producing genes were used for Z-score, and then 203 tumor samples in the HCCDB18 dataset were grouped into subtypes. Like TCGA, they were also divided into four groups: Quiescent group, Glycolysis group, Cholesterol group, and Mixed group (Figure 4(a)). Further, the prognostic relationship between the two groups was analyzed. In all datasets, the prognosis of the Mixed group was the worst, while the Quiescent group had a good prognosis (Figure 4(b), log-rank*P* < 0.01), which was the same as that of TCGA. The expression levels of glycolysis/cholesterol synthesis-related genes in the four groups were different (Figure 4(c)).

The frequency of commonly mutated genes was also studied in LIHC between SNV/InDel and CNV affecting molecular subtypes in the HCCDB18 dataset (Figure 4(d)). The results showed that the type and number of mutations were lower than those in the TCGA dataset, but it was also found that TP53 mutation and CTNNB1 mutation were mutually exclusive, and no significant difference was seen in the mutation frequency of each gene between different groups. According to the expression of cholesterol and glycolytic co-expression genes, the datasets of TCGA and HCCDB18 were metabolically typed. It was found that the prognosis of the Mixed group was the worst in both datasets, while the Quiescent group had a good prognosis, which showed that for liver cancer samples, high cholesterol genes plus high expression glycolytic genes would lead to poor prognosis. Low expression of glycolysis/cholesterol synthesis-associated genes would lead to a better prognosis.

### 3.5. Identification of DEGs

For identification of the role of glycolysis/cholesterol axis genes in LIHC tumors, DEGs were found in the datasets TCGA and HCCDB18, respectively. We also performed a functional enrichment analysis. For the TCGA dataset, 507 DEGs were identified between Mixed and Quiescent groups, including 431 enriched and 76 depleted genes. The results revealed that the major enriched DEGs were between the Mixed and Quiescent groups (Supplementary Figure S2(a)). The clustering of up- and down-regulated genes showed that they were obviously clustered into two categories. The genes up-regulated in Mixed were down-regulated in Quiescent, and the genes up-regulated in Quiescent were down-regulated in Mixed (Supplementary Figure S2(b)). For the HCCDB18 dataset, 261 differential genes were identified between Mixed and Quiescent, including 159 enriched genes and 102 depleted genes. Similar to the results of TCGA, the results of the HCCDB18 dataset showed that Mixed and Quiescent groups mainly included up-regulated DEGs (Supplementary Figure S2(c)), and the clustering of enriched and depleted DEGs also showed that they were obviously clustered into two categories (Supplementary Figure S2(d)).

**Figure 5:**
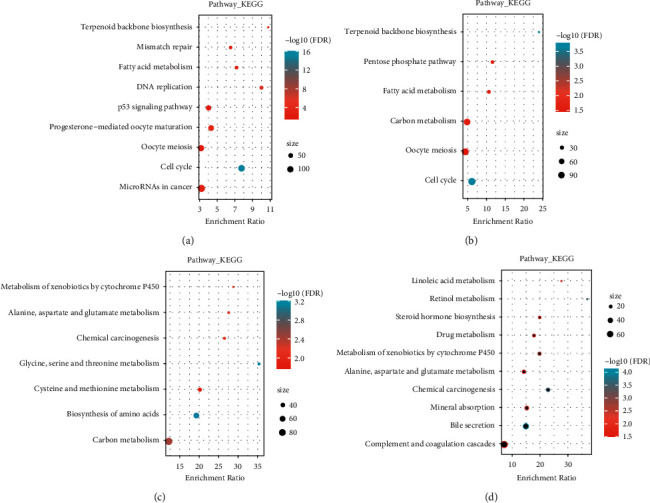
KEGG analysis of DEGs. (a-b) KEGG annotation of upregulated DEGs in Mixed group in TCGA (a) and HCCDB18 (b) datasets; (c-d) KEGG annotation of downregulated DEGs in Mixed group in TCGA (c) and HCCDB18 (d) datasets.

Further, KEGG pathway analysis was conducted on the enriched DEGs between the Mixed and Quiescent groups in TCGA and HCCDB18, respectively. For the up-regulated DEGs, nine and six significant pathways were annotated in TCGA and HCCDB18 datasets, respectively (Figures 5(a) and 5(b), FDR <0.05). It was found that the genes up-regulated in the Mixed group were significantly related to tumorigenesis and metabolic pathways such as the P53 signaling pathway, microRNAs in cancer, fatty acid metabolism, pentose phosphate pathway, carbon metabolism, cell cycle, and DNA replication. For the down-regulated DEGs between the Mixed group and the Quiescent group, we observed that metabolic pathways were also significantly enriched such as the metabolism of xenobiotics by cytochrome P450, alanine, aspartate and glutamate metabolism, carbon metabolism, and drug metabolism (FDR < 0.05, Figures 5(c) and 5(d)).

### 3.6. Pathway Analysis in Different Molecular Subtypes

**Figure 6:**
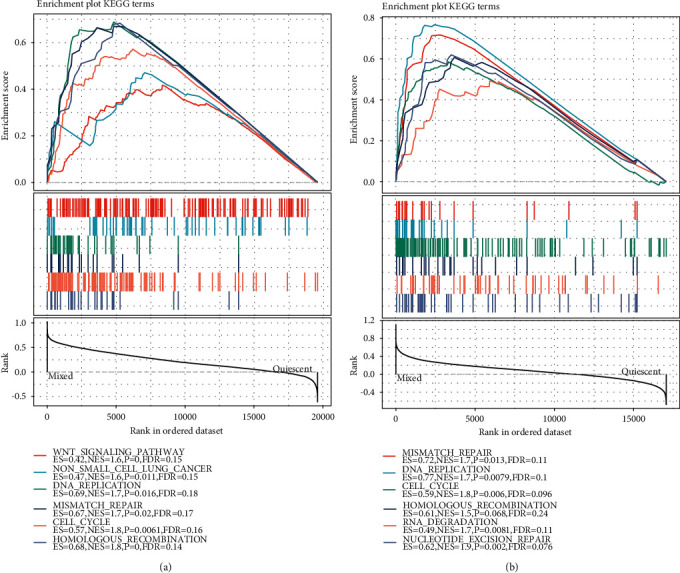
GSEA of molecular subtypes. (a) GSEA results of Mixed and Quiescent groups in TCGA dataset. (b) GSEA results of Mixed and Quiescent groups in HCCDB18 dataset.

The significantly enriched pathways in the Mixed and Quiescent groups were analyzed using GSEA in TCGA and HCCDB18 datasets (Figure 6). It was found that the WNT signaling pathway, DNA replication, mismatch repair, cell cycle, and homologous recombination related to tumor incidence and development were significantly enriched in the Mixed group of TCGA and HCCDB18 datasets. This phenomenon was consistent with the functional enrichment results of differential genes between the Mixed group and the Quiescent group. At the same time, it showed that the high expression of glycolysis/cholesterol synthesis-related genes in liver cancer was associated with a poorer prognosis.

### 3.7. Comparison of Glycolysis and Cholesterol Gene Expression in Normal and Tumor Samples

**Figure 7:**
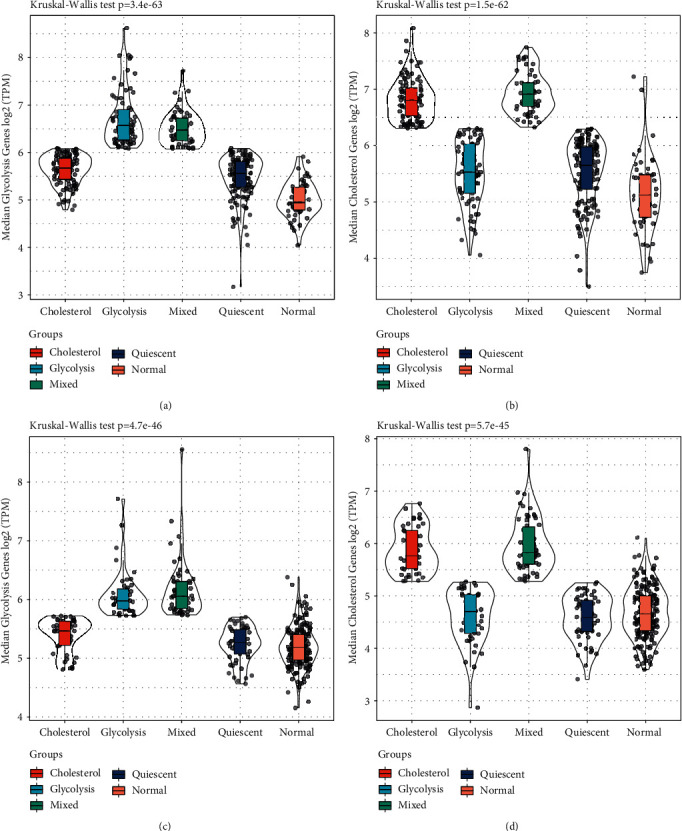
Comparative analysis of glycolysis/cholesterol synthesis-related gene samples. (a) The expression level of glycolysis-related genes among different groups in TCGA dataset. (b) Cholesterol synthesis-related genes expression levels among different groups in TCGA dataset. (c) The expression level of glycolysis-related genes among different groups in HCCDB18 dataset. (d) Cholesterol synthesis-related genes expression levels among different groups in HCCDB18 dataset.

In order to compare the expression of these glycolytic and cholesterol synthesis-related genes in normal and tumor samples, their expression profiles were extracted, and the median was used to compare them in different tumor sample subgroups and normal samples (Figure 7). The results showed that in the groups of TCGA and HCCDB18 datasets, the expression levels of cholesterol gene and glycolytic gene in normal samples were lower than those of the other four molecular subtypes, which also showed that the incidence and development of liver cancer would be associated to the increase of glycolytic and cholesterol synthetic gene expression.

### 3.8. Comparison of Clinical Characteristics between Different Molecular Subtypes

In the TCGA dataset, the distribution of different clinical features was compared in the four molecular subtypes and whether the clinical features were different in different groups (Supplementary Figure S3(a)–S3(h)). The results displayed no difference in the distribution of age, gender, recurrence, N stage, and *M* stage among the four molecular subtypes. Regarding grade classification, there was a significant difference between the Mixed group and the Quiescent group; G2 accounted for the smallest proportion in the Mixed group, while G3 and G4 accounted for the largest proportion. Also, significant differences were observed between the Glycolysis group and the other three molecular subtypes in the characteristics of *T* Stage and Stage staging. Among them, for *T* Stage, T1 had the smallest proportion in the Glycolysis group, and T2 had the largest proportion in the Glycolysis group; For Stage I-IV, Stage I had the smallest proportion in the Glycolysis group, and Stage II and Stage IV had the largest proportions in the Glycolysis group.

Similarly, in the HCCDB18 dataset, different clinical features' distribution in four molecular subtypes were compared to see whether there were differences in clinical features in different groups (Supplementary Figures S3(i) and S3(j)). The results displayed no significant difference in the distribution of age and gender among the four molecular subtypes. The Mixed group showed a significant difference from the Cholesterol and Quiescent groups in terms of the characteristics of *T* Stage.

### 3.9. Comparing Our Molecular Subtypes and Existing Immune Molecular Subtypes

Previous studies have found a relationship between glycolytic/cholesterol metabolism and immune infiltration [35, 36]. Therefore, we compared the distribution of our molecular subtypes to the previous immune subtypes. Six immune infiltration types were identified in human tumors from counterpart tumor promotion to tumor suppressors, including C1 (wound healing), C2 (INF-r dominant), C3 (inflammation), C4 (lymphocyte depletion), C5 (immunological silencing), and C6 (TGF-beta dominant), among which, C1, C2, and C6 showed the poorest prognoses [37]. Most HCC patients in the TCGA data of LIHC were categorized into C3 and C4 immune subtypes (about 80%), one patient was categorized as C6, and no patient was posited in the C5 immune subtype. It is noteworthy that the proportion of C1 and C2 immune subtypes in the Mixed and Glycolysis groups was revealed to be significantly higher compared to that in the Quiescent and Cholesterol groups. The immune subtype distribution in different metabolic groups showed significant differences (Supplementary Figure S4).

### 3.10. Comparison of Immune Scores between Different Molecular Subtypes

**Figure 8:**
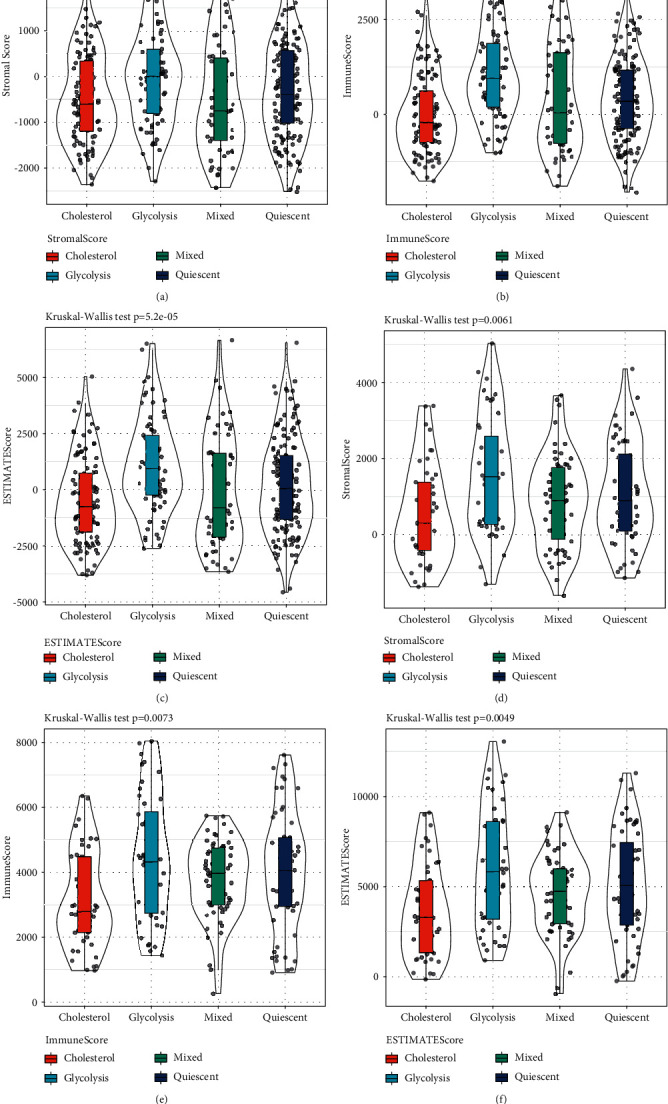
Comparison of immune scores between molecular subtypes. A-C: comparison of immune scores between molecular subtypes in TCGA dataset. D-F: comparison of immune scores between molecular subtypes in HCCDB18 dataset.

In order to identify the relationship between the immune and matrix scores of the four molecular subtypes, the immune and matrix scores of each sample were first calculated using the R software package estimate, and then they were compared. The results displayed significant differences in the four metabolic groups in the TCGA and HCCDB18 datasets StromalScore, ImmuneScore, and ESTIMATEScore (Figure 8, *P* < 0.01). StromalScore and ImmuneScore were significantly higher in the Glycolysis group compared to the other three groups, and ImmuneScore in the Cholesterol group was significantly lower than in the other three groups. In addition, we performed CIBERSORT to assess the distribution of 22 immune cells in four molecular subtypes. Nine immune cells were differentially distributed in four subtypes, including resting and activated memory CD4 *T* cells, resting natural killer (NK) cells, monocytes, M0 macrophages, M2 macrophages, resting and activated mast cells, and eosinophils (Supplementary Figure S5).

### 3.11. Single Factor and Multi-Factor Analysis

The related HR, 95% CI of HR, and *P* value of clinical characteristics and molecular subtypes were analyzed using univariate Cox regression analysis on the clinical information obtained from the whole TCGA dataset. Our molecular subtype (Table 1) and the clinical information recorded by TCGA for patients, including age, gender, *T* stage, stage, grade, and recurrence status. It could be seen that the risk of *T*3 + *T*4 in *T* Stage staging was higher than that of *T*1 + *T*2 staging samples; the risk of Stage III + IV samples was higher than that of I + II samples; the risk of Quiescent group samples was lower than that of other subtypes, while the risk of Mixed group was higher than that of other groups. At the same time, the multivariate Cox analysis of these clinical features and molecular subtypes was analyzed (Table 2). The results showed that the Mixed group could still be an independent prognostic factor in the multivariate analysis, indicating the reliability of our grouping.

## 4. Discussion

The research on clinical-related tumor molecular subtypes needs to move forward to accelerate the development of personalized treatment of liver cancer. According to the expression profiles, this study showed that genes could be clustered as 44 cholesterol/glycolysis-related genes, including 13 glycolysis co-clustering genes and 17 cholesterol co-polymerization genes. According to the expression of these genes, the samples of the TCGA dataset were categorized into four metabolic groups (Quiescent, Glycolysis, Cholesterol, and Mixed). Survival analysis revealed that the Mixed group had a poorer prognosis, while the Quiescent group had a better prognosis. Similarly, these genes were used to classify the metabolism of the independent HCCDB18 dataset, which was also divided into four groups, and the same was that the Mixed group has a poor prognosis, while the Quiescent group had a better prognosis. This suggested that the high expression of cholesterol/glycolysis-related genes in hepatocellular carcinoma was associated with poor prognosis.

Studies on molecular typing of tumors based on the glycolysis-cholesterol synthesis axis are relatively rare, and there are no reports of liver cancer at present, but glycolysis can promote tumor progression, immune escape, and chemical resistance [12], and some studies show that glycolysis related gene expression profile can be used as a new prognostic risk predictor of human hepatocellular carcinoma [38, 39]. At the same time, cholesterol and its associated metabolites induce the growth of cancer cells [40], while the AMPK anti-cancer function is partially mediated through sterol synthesis inhibition [41]. Studies in liver cancer have shown that some genes related to cholesterol metabolism can provide candidate targets for its differential diagnosis [42]. Therefore, this article provides a new perspective for the study of liver cancer based on the typing of the glycolysis-cholesterol synthesis axis. Compared with the existing immune molecular subtypes, it was found that the Mixed group contained more immune subtypes C1 and C2 with poor prognosis, which showed that our typing was reasonable and reliable.

Therefore, the typing proposed in this study was a meaningful new typing, and it was found that the differentially up-regulated genes in the Mixed group in TCGA and HCCDB18 dataset were related to the pathway of tumorigenesis and development, whereas the depleted DEGs related to the metabolism. At the same time, GSEA analysis in TCGA and HCCDB18 datasets also revealed that the Mixed group samples were significantly correlated with tumor-related pathways. Cancer cells enhanced carcinogenic metabolic pathways such as glutamine metabolism, pentose phosphate pathway, fatty acid synthesis, and cholesterol synthesis. This carcinogenic metabolism involved several transcription factors and molecules, including WNT [43]. Therefore, the Wnt signaling pathway enriched in the Mixed group in our study was consistent with the study that Wnt could regulate carcinogenic metabolism and contribute to cell invasion and metastasis.

Tumor metabolic heterogeneity has been attributed to mutations in somatic driver genes as well as tumor subtypes [13]. The Loss ratio of the TP53 gene and the Gain ratio of CTNNB1 were significantly higher in the Mixed group compared to the Quiescent group. Carcinogenic TP53 and CTNNB1 mutations with loss of function have been reported to have an inducing effect on the glycolysis pathway in cancer. Moreover, glycolysis has been shown to contribute to tumor progression and chemotherapy resistance in tumors [14, 15]. The present findings showed to be consistence with earlier studies reported in the literature, indicating the reliability of our molecular subtype. In addition, decreased MPC activity has been revealed to be associated with a poorer prognosis in some cancer types [18].

In the four groups of the TCGA dataset, the mutation and copy number variations in the four groups were compared. Accordingly, (1) no significant difference in SNV/InDel was found among the four groups; (2) the proportion of Loss of gene TP53 was significantly higher in the Mixed group compared to that in the Quiescent group; and (3) the proportion of Gain genes MYC and CTNNB1 was significantly higher in the Mixed group compared to that in the Quiescent group.

According to the present findings, it was found that the Glycolytic group had higher immune infiltration and might respond better the immunotherapy. This study proved that the mutation in a variety of metabolic genes and the expression of specific enzymes led to the unique metabolic profile that can be used to predict the clinical prognosis of specific liver cancers. Metabolic analysis of hepatocellular carcinoma based on the metabolic reprogramming that occurred in the cancerous cells can be used as constructive guidelines for determining the treatment alternatives, drug resistance possibility, as well as predicting the expecting responses and potential treatment outcomes.

Although many sufficient analyses were made in the early stage of this study, by comparing the expression profile of glycolysis/cholesterol synthesis-related genes in different groups, we observed a significantly higher expression of molecular subtype compared to that of normal samples, which confirms the certain relationship of incidence and progression of liver cancer with the glycolysis/cholesterol-related genes. However, further experimental evidence is still needed to confirm the conclusions of this study, such as experimental verification of the differences in the expression profiles of glycolysis/cholesterol-related genes, exploring significant differences in the protein expression levels of DEGs, and the key effects of these DEGs on tumor progression and prognosis, in different subtypes. Additionally, the possible interaction regulation mechanism needs to be further studied.

The molecular subtype identified in this paper show different prominent clinical characteristics, mutation characteristics, pathway characteristics and immune characteristics, which provide guidance for the research of targeted drugs based on the glycolysis cholesterol synthesis axis. Different groups of patients with liver cancer should be evaluated in clinical trials, and this classification can be used as an important auxiliary means of histopathology. Through the existing means of tissue detection, observation, and determination, as well as the use of emerging genome detection to query the mutation and amplification of key genomes, the taxonomic subtypes developed in the present study can be applied to new cases of liver cancer. It is expected that the present results will promote the development of clinical trials, explore treatment methods in the identified patient groups, and finally improve the survival rate of this fatal disease.

## 5. Conclusion

Based on the glycolysis-cholesterol synthesis axis, liver cancer was stratified into four metabolic groups (Quiescent, Glycolysis, Cholesterol, and Mixed) through two liver cancer datasets, and significant differences were observed in the prognosis of the groups. Among them, the Mixed group had a poor prognosis, whereas the Quiescent group had a better prognosis. This suggested that the high expression of glycolysis/cholesterol synthesis-related genes in hepatocellular carcinoma was associated with poor prognosis.

## Figures and Tables

**Table 1:** Univariate Cox regression analysis of clinical features and molecular subtypes in TCGA dataset.

Variables	Univariable analysis			
	HR	95% CI of HR		*P*
		Lower	Upper	
Age				
≤65				
>65	1.27	0.89	1.79	0.18

Gender				
Female				
Male	0.82	0.57	1.16	0.26

*T* Stage				
*T*1 + *T*2				
*T*3 + *T*4	2.54	1.79	3.61	**2.2E-07**

Stage				
I + II				
III + IV	2.45	1.69	3.55	**2.3E-06**

Grade				
*G*1 + *G*2				
*G*3 + *G*4	1.12	0.78	1.61	0.54

Recurrence				
NO				
YES	1.24	0.87	1.76	0.23

Subtype				
Other				
Cholesterol	0.65	0.43	0.98	**0.04**

Subtype				
Other				
Glycolysis	1.54	1.04	2.29	**0.03**

Subtype				
Other				
Mixed	2.69	1.76	4.10	**4.7E-06**

Subtype				
Other				
Quiescent	0.62	0.42	0.90	**0.01**

**Table 2:** Multivariate Cox regression analysis of clinical features and molecular subtypes in TCGA dataset.

Variables	Multivariable analysis			
	HR	95% CI of HR		*P*
		Lower	Upper	
*Cholesterol*				
Age	1.28	0.88	1.88	0.20
Gender	0.84	0.57	1.23	0.37
T Stage	2.64	0.35	19.80	0.34
Stage	1.00	0.14	7.45	1.00
Grage	1.17	0.80	1.71	0.41
Recurrence	1.06	0.72	1.54	0.78
Cholesterol	0.56	0.36	0.88	**0.01**

*Glycolysis*				
Age	1.25	0.86	1.84	0.25
Gender	0.85	0.58	1.26	0.43
T Stage	2.37	0.32	17.85	0.40
Stage	1.05	0.14	7.85	0.96
Grage	1.17	0.80	1.71	0.42
Recurrence	1.07	0.73	1.56	0.73
Glycolysis	1.36	0.87	2.11	0.18

*Mixed*				
Age	1.21	0.82	1.78	0.33
Gender	0.81	0.55	1.19	0.28
T Stage	3.33	0.44	25.19	0.24
Stage	0.73	0.10	5.54	0.76
Grage	0.98	0.66	1.46	0.91
Recurrence	0.99	0.68	1.45	0.97
Mixed	2.77	1.74	4.42	**1.7E-05**

*Quiescent*				
Age	1.22	0.83	1.79	0.31
Gender	0.84	0.57	1.23	0.37
T Stage	2.30	0.31	17.17	0.42
Stage	1.05	0.14	7.82	0.96
Grage	1.13	0.77	1.66	0.53
Recurrence	1.06	0.72	1.55	0.77
Quiescent	0.74	0.49	1.11	0.14

## Data Availability

The datasets used and/or analyzed during the current study are available from the corresponding author on reasonable request. TCGA-LIHC dataset was accessed from the website https://portal.gdc.cancer.gov/projects/TCGA-LIHC. HCCDB18 dataset was accessed from the website http://lifeome.net/database/hccdb/download.html.

## Supplementary Materials

Supplementary Materials Supplementary Figure S1. Comparison between molecular subtype and existing immune molecular subtypes in TCGA dataset. A: Comparison between molecular subtype and existing immune subtypes. B: Distribution comparison of immune subtypes among different molecular subtypes. Supplementary Figure S2. GO functional annotation of MPC1/2 related genes in TCGA dataset. ABC: GO function annotation of genes positively related to MPC1/2. DE: GO function annotation of genes negatively related to MPC1/2. Supplementary Figure S3. Identification and analysis of differentially expressed genes. A: Volcano map of DEGs between Mixed and Quiescent groups in TCGA dataset. B: Heatmap of differentially grouped genes in TCGA dataset mixed and quiet. C: Volcano map of DEGs in Mixed and Quiescent groups of HCCDB18 dataset. D: Heatmap of differentially grouped genes in the HCCDB18 dataset of Mixed and Quiescent groups. Supplementary Figure S4. (A-H) Distribution comparison of different clinical characteristics among four molecular subtypes in TCGA dataset. (I–K) Distribution comparison of different clinical features among four molecular subtypes in the HCCDB18 dataset. Supplementary Figure S5. The estimated distribution of 22 immune cells in four molecular subtypes in TCGA dataset. ns, no significance. ^*∗*^*P* < 0.05, ^*∗∗*^*P* < 0.01, ^*∗∗∗*^*P* < 0.001, ^*∗∗∗∗*^*P* < 0.0001. Supplementary Table S1. A list of 44 glycolysis/cholesterol-related genes.
