# Supplementary material for: Classification and Prognostic Characteristics of Hepatocellular Carcinoma Based on Glycolysis Cholesterol Synthesis Axis
Source: J Oncol. 2022 Sep 30;2022:2014625. doi: 10.1155/2022/2014625 (PMC9546679; doi:10.1155/2022/2014625)
Supplement: Supplementary Materials — Supplementary Figure S1. Comparison between molecular subtype and existing immune molecular subtypes in TCGA dataset. A: Comparison between molecular subtype and existing immune subtypes. B: Distribution comparison of immune subtypes among different molecular subtypes. Supplementary Figure S2. GO functional annotation of MPC1/2 related genes in TCGA dataset. ABC: GO function annotation of genes positively related to MPC1/2. DE: GO function annotation of genes negatively related to MPC1/2. Supplementary Figure S3. Identification and analysis of differentially expressed genes. A: Volcano map of DEGs between Mixed and Quiescent groups in TCGA dataset. B: Heatmap of differentially grouped genes in TCGA dataset mixed and quiet. C: Volcano map of DEGs in Mixed and Quiescent groups of HCCDB18 dataset. D: Heatmap of differentially grouped genes in the HCCDB18 dataset of Mixed and Quiescent groups. Supplementary Figure S4. (A-H) Distribution comparison of different clinical characteristics among four molecular subtypes in TCGA dataset. (I–K) Distribution comparison of different clinical features among four molecular subtypes in the HCCDB18 dataset. Supplementary Figure S5. The estimated distribution of 22 immune cells in four molecular subtypes in TCGA dataset. ns, no significance. ∗P < 0.05, ∗∗P < 0.01, ∗∗∗P < 0.001, ∗∗∗∗P < 0.0001. Supplementary Table S1. A list of 44 glycolysis/cholesterol-related genes. [file 2014625.f1.zip › Supplementary Figure S1.pdf]

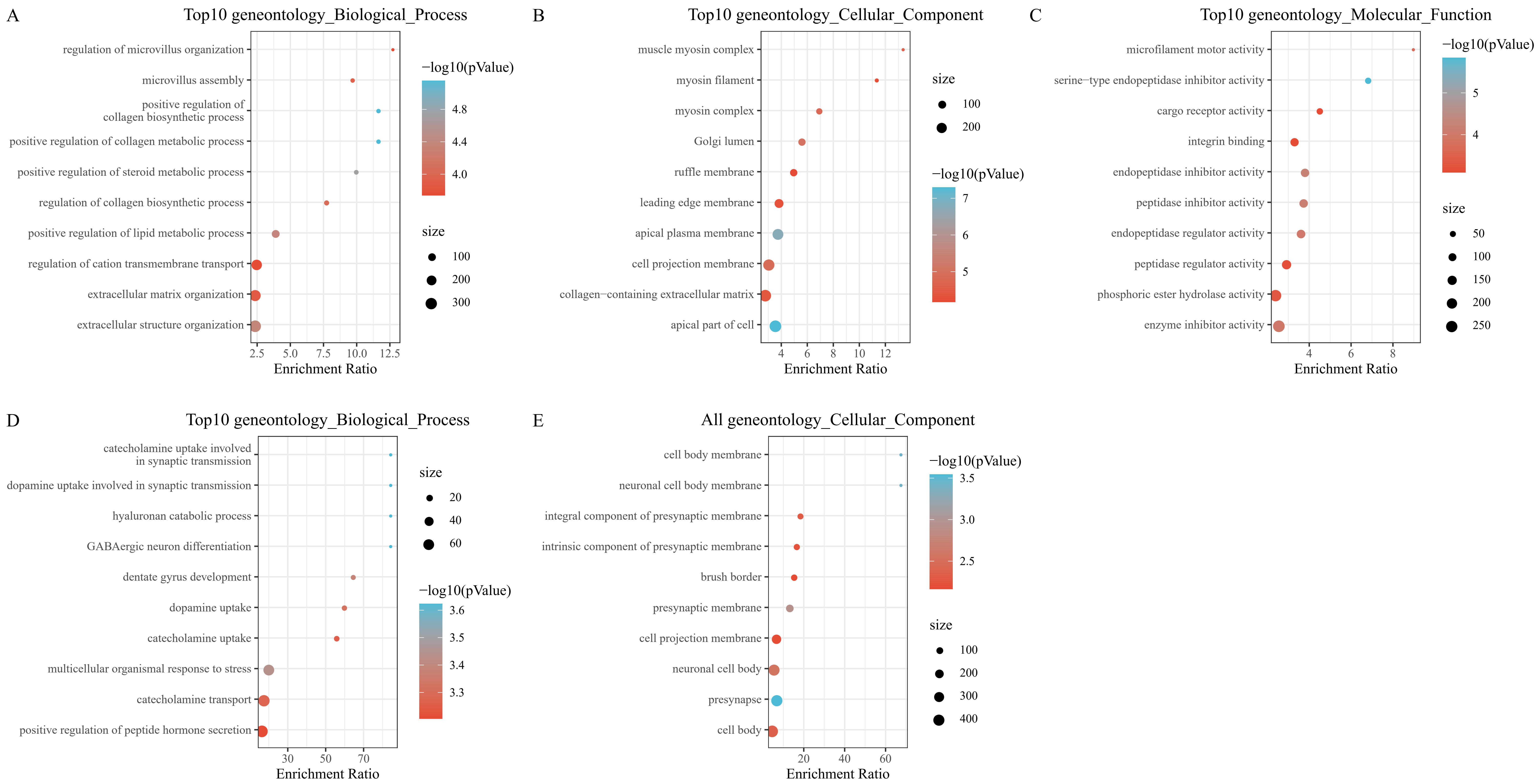
A
Top10 geneontology_Biological_Process
regulation of microvillus organization
microvillus assembly
positive regulation of collagen biosynthetic process
positive regulation of collagen metabolic process
positive regulation of steroid metabolic process
regulation of collagen biosynthetic process
positive regulation of lipid metabolic process
regulation of cation transmembrane transport
extracellular matrix organization
extracellular structure organization
2.5 5.0 7.5 10.0 12.5
Enrichment Ratio
−log10(pValue)
4.8
4.4
4.0
size
100
200
300
B
Top10 geneontology_Cellular_Component
muscle myosin complex
myosin filament
myosin complex
Golgi lumen
ruffle membrane
leading edge membrane
apical plasma membrane
cell projection membrane
collagen−containing extracellular matrix
apical part of cell
4 6 8 10 12
Enrichment Ratio
size
100
200
−log10(pValue)
7
6
5
C
Top10 geneontology_Molecular_Function
microfilament motor activity
serine−type endopeptidase inhibitor activity
cargo receptor activity
integrin binding
endopeptidase inhibitor activity
peptidase inhibitor activity
endopeptidase regulator activity
peptidase regulator activity
phosphoric ester hydrolase activity
enzyme inhibitor activity
4 6 8
Enrichment Ratio
−log10(pValue)
5
4
size
50
100
150
200
250
D
Top10 geneontology_Biological_Process
catecholamine uptake involved in synaptic transmission
dopamine uptake involved in synaptic transmission
hyaluronan catabolic process
GABAergic neuron differentiation
dentate gyrus development
dopamine uptake
catecholamine uptake
multicellular organismal response to stress
catecholamine transport
positive regulation of peptide hormone secretion
30 50 70
Enrichment Ratio
size
20
40
60
−log10(pValue)
3.6
3.5
3.4
3.3
E
All geneontology_Cellular_Component
cell body membrane
neuronal cell body membrane
integral component of presynaptic membrane
intrinsic component of presynaptic membrane
brush border
presynaptic membrane
cell projection membrane
neuronal cell body
presynapse
cell body
20 40 60
Enrichment Ratio
−log10(pValue)
3.5
3.0
2.5
size
100
200
300
400
