# Supplementary material for: Classification and Prognostic Characteristics of Hepatocellular Carcinoma Based on Glycolysis Cholesterol Synthesis Axis
Source: J Oncol. 2022 Sep 30;2022:2014625. doi: 10.1155/2022/2014625 (PMC9546679; doi:10.1155/2022/2014625)
Supplement: Supplementary Materials — Supplementary Figure S1. Comparison between molecular subtype and existing immune molecular subtypes in TCGA dataset. A: Comparison between molecular subtype and existing immune subtypes. B: Distribution comparison of immune subtypes among different molecular subtypes. Supplementary Figure S2. GO functional annotation of MPC1/2 related genes in TCGA dataset. ABC: GO function annotation of genes positively related to MPC1/2. DE: GO function annotation of genes negatively related to MPC1/2. Supplementary Figure S3. Identification and analysis of differentially expressed genes. A: Volcano map of DEGs between Mixed and Quiescent groups in TCGA dataset. B: Heatmap of differentially grouped genes in TCGA dataset mixed and quiet. C: Volcano map of DEGs in Mixed and Quiescent groups of HCCDB18 dataset. D: Heatmap of differentially grouped genes in the HCCDB18 dataset of Mixed and Quiescent groups. Supplementary Figure S4. (A-H) Distribution comparison of different clinical characteristics among four molecular subtypes in TCGA dataset. (I–K) Distribution comparison of different clinical features among four molecular subtypes in the HCCDB18 dataset. Supplementary Figure S5. The estimated distribution of 22 immune cells in four molecular subtypes in TCGA dataset. ns, no significance. ∗P < 0.05, ∗∗P < 0.01, ∗∗∗P < 0.001, ∗∗∗∗P < 0.0001. Supplementary Table S1. A list of 44 glycolysis/cholesterol-related genes. [file 2014625.f1.zip › Supplementary Table S1 (2).pdf]

Supplementary Table S1. A list of 44 glycolysis/cholesterol-related genes.

	Genes	Type
ALDOA	ALDOA	GLYCOLYSIS
ENO1	ENO1	GLYCOLYSIS
ENO3	ENO3	GLYCOLYSIS
DHCR7	DHCR7	CHOLESTEROL
MVK	MVK	CHOLESTEROL
CYP51A1	CYP51A1	CHOLESTEROL
DHCR24	DHCR24	CHOLESTEROL
PGK1	PGK1	GLYCOLYSIS
PFKM	PFKM	GLYCOLYSIS
PKLR	PKLR	GLYCOLYSIS
PPP2CA	PPP2CA	GLYCOLYSIS
ALDOB	ALDOB	GLYCOLYSIS
GAPDH	GAPDH	GLYCOLYSIS
EBP	EBP	CHOLESTEROL
TM7SF2	TM7SF2	CHOLESTEROL
MVD	MVD	CHOLESTEROL
MSMO1	MSMO1	CHOLESTEROL
PFKFB2	PFKFB2	GLYCOLYSIS
HMGCS1	HMGCS1	CHOLESTEROL
TPI1	TPI1	GLYCOLYSIS
GGPS1	GGPS1	CHOLESTEROL
SQLE	SQLE	CHOLESTEROL
ENO2	ENO2	GLYCOLYSIS
PFKL	PFKL	GLYCOLYSIS
HSD17B7	HSD17B7	CHOLESTEROL
PPP2R5D	PPP2R5D	GLYCOLYSIS
PFKFB1	PFKFB1	GLYCOLYSIS
PFKFB3	PFKFB3	GLYCOLYSIS
HMGCR	HMGCR	CHOLESTEROL
GPI	GPI	GLYCOLYSIS
FDFT1	FDFT1	CHOLESTEROL
PMVK	PMVK	CHOLESTEROL
PPP2R1B	PPP2R1B	GLYCOLYSIS
PPP2R1A	PPP2R1A	GLYCOLYSIS
IDI1	IDI1	CHOLESTEROL
LBR	LBR	CHOLESTEROL
ALDOC	ALDOC	GLYCOLYSIS
LSS	LSS	CHOLESTEROL
PGAM1	PGAM1	GLYCOLYSIS
PFKP	PFKP	GLYCOLYSIS
NSDHL	NSDHL	CHOLESTEROL
PPP2CB	PPP2CB	GLYCOLYSIS
FDPS	FDPS	CHOLESTEROL
TPI1P1	TPI1P1	GLYCOLYSIS
