# Supplementary figures and images for: Classification and Prognostic Characteristics of Hepatocellular Carcinoma Based on Glycolysis Cholesterol Synthesis Axis
Source: J Oncol. 2022 Sep 30;2022:2014625. doi: 10.1155/2022/2014625 (PMC9546679; doi:10.1155/2022/2014625)

A

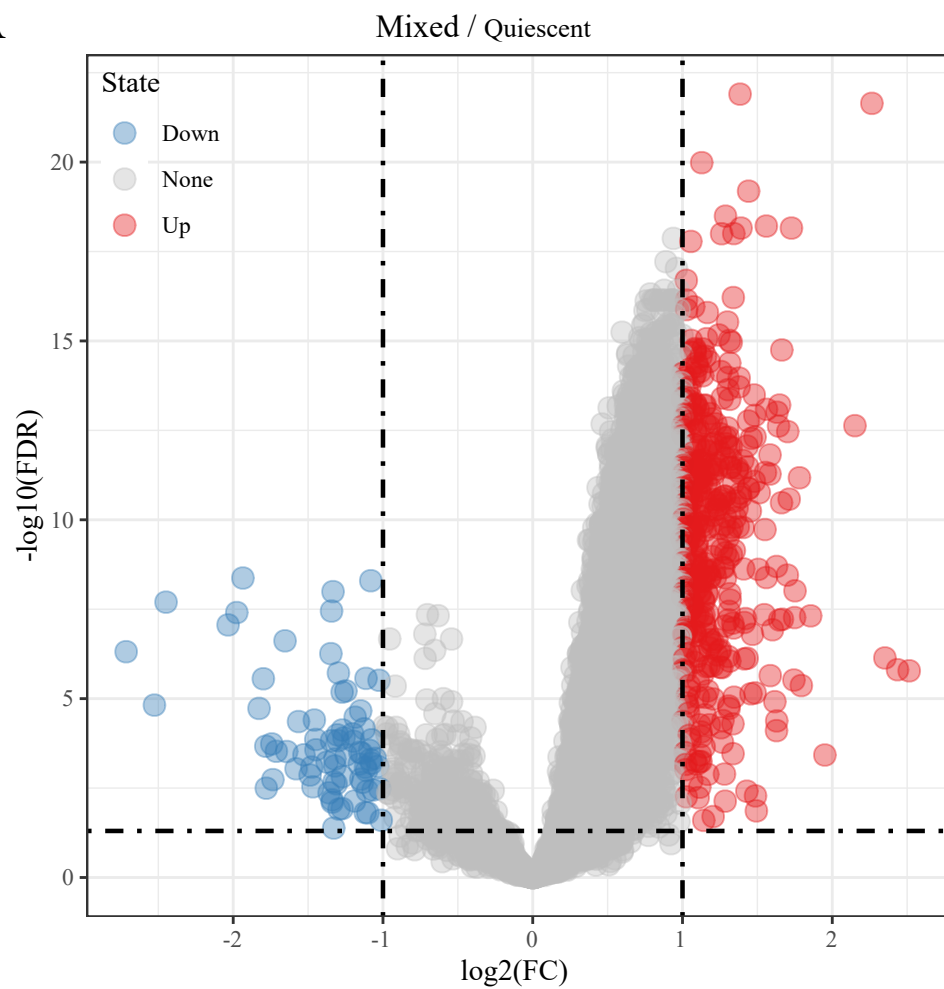

B

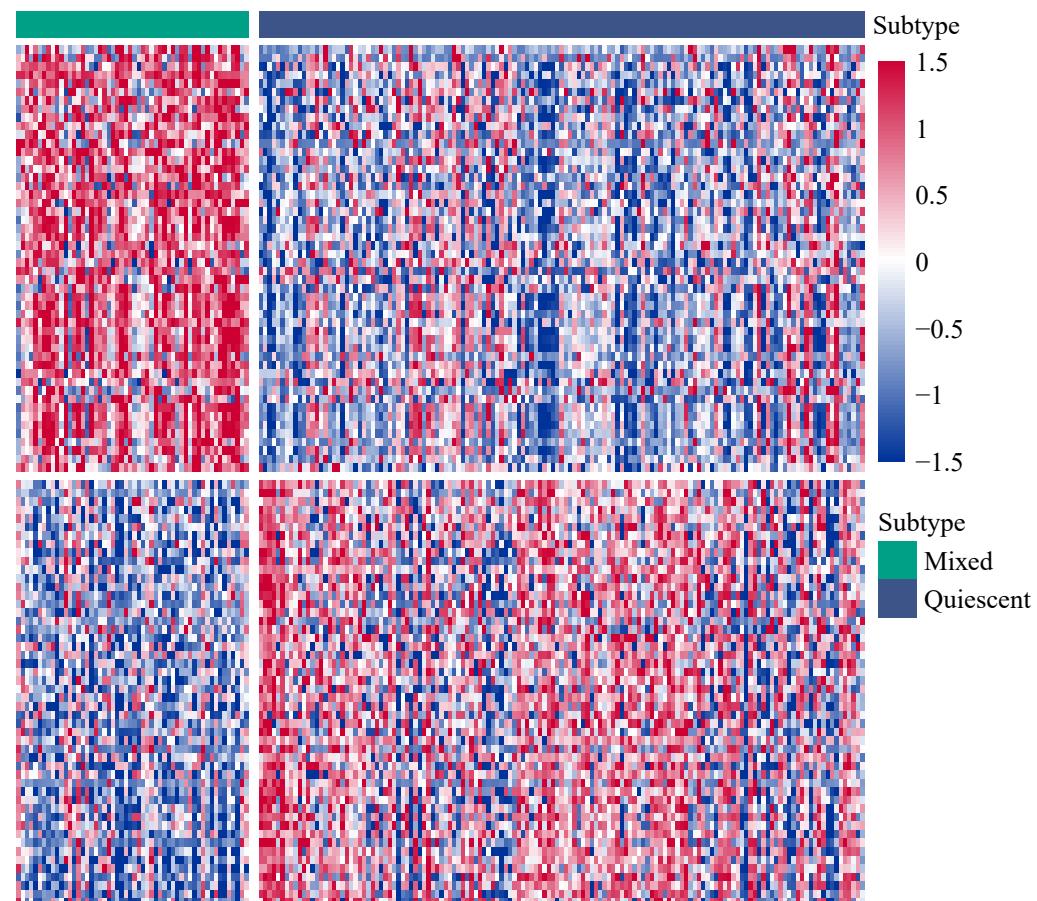

C

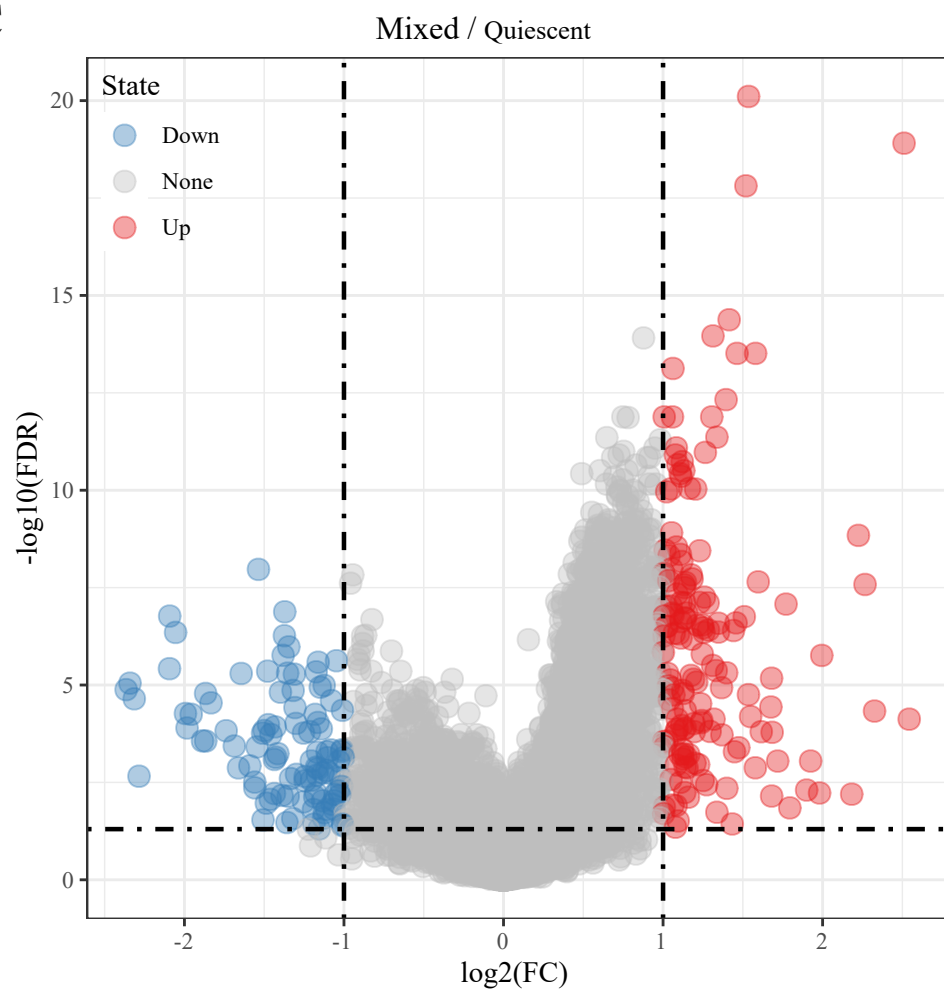

D

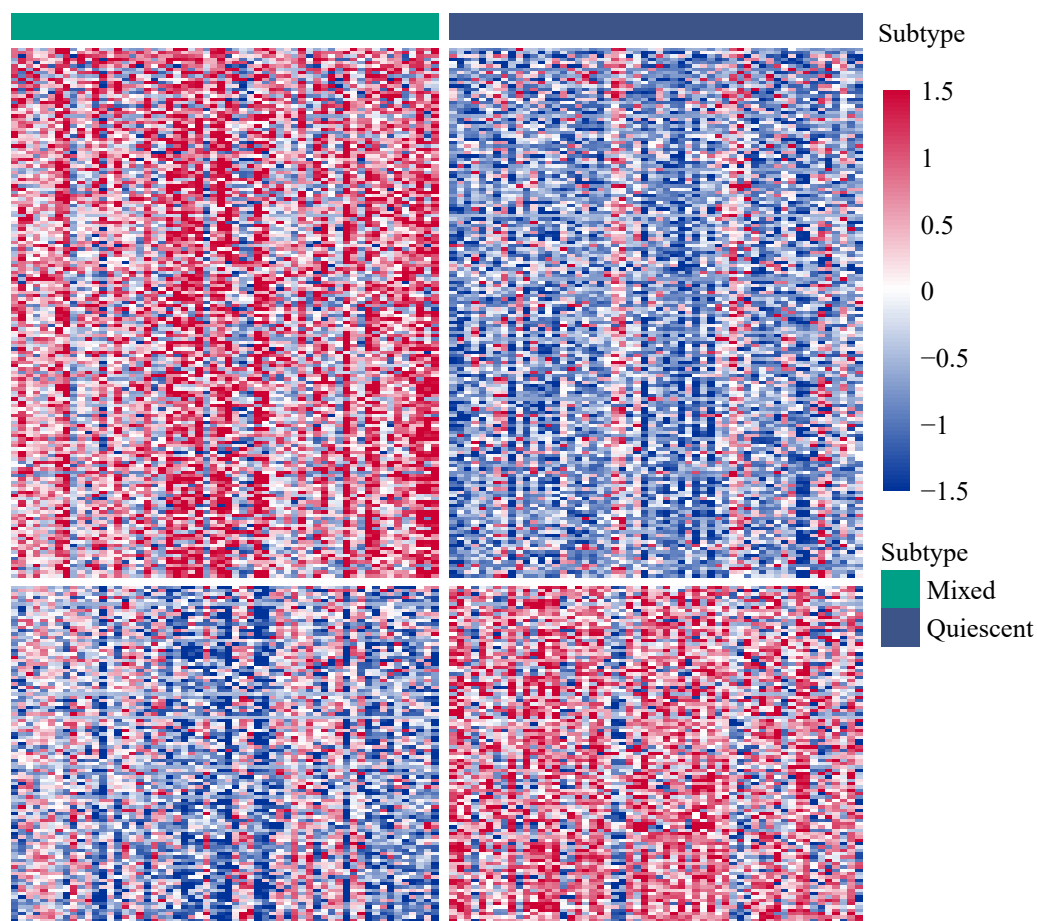

Supplement: Supplementary Materials — Supplementary Figure S1. Comparison between molecular subtype and existing immune molecular subtypes in TCGA dataset. A: Comparison between molecular subtype and existing immune subtypes. B: Distribution comparison of immune subtypes among different molecular subtypes. Supplementary Figure S2. GO functional annotation of MPC1/2 related genes in TCGA dataset. ABC: GO function annotation of genes positively related to MPC1/2. DE: GO function annotation of genes negatively related to MPC1/2. Supplementary Figure S3. Identification and analysis of differentially expressed genes. A: Volcano map of DEGs between Mixed and Quiescent groups in TCGA dataset. B: Heatmap of differentially grouped genes in TCGA dataset mixed and quiet. C: Volcano map of DEGs in Mixed and Quiescent groups of HCCDB18 dataset. D: Heatmap of differentially grouped genes in the HCCDB18 dataset of Mixed and Quiescent groups. Supplementary Figure S4. (A-H) Distribution comparison of different clinical characteristics among four molecular subtypes in TCGA dataset. (I–K) Distribution comparison of different clinical features among four molecular subtypes in the HCCDB18 dataset. Supplementary Figure S5. The estimated distribution of 22 immune cells in four molecular subtypes in TCGA dataset. ns, no significance. ∗P < 0.05, ∗∗P < 0.01, ∗∗∗P < 0.001, ∗∗∗∗P < 0.0001. Supplementary Table S1. A list of 44 glycolysis/cholesterol-related genes. [file 2014625.f1.zip › Supplementary Figure S2 (1).pdf]

A

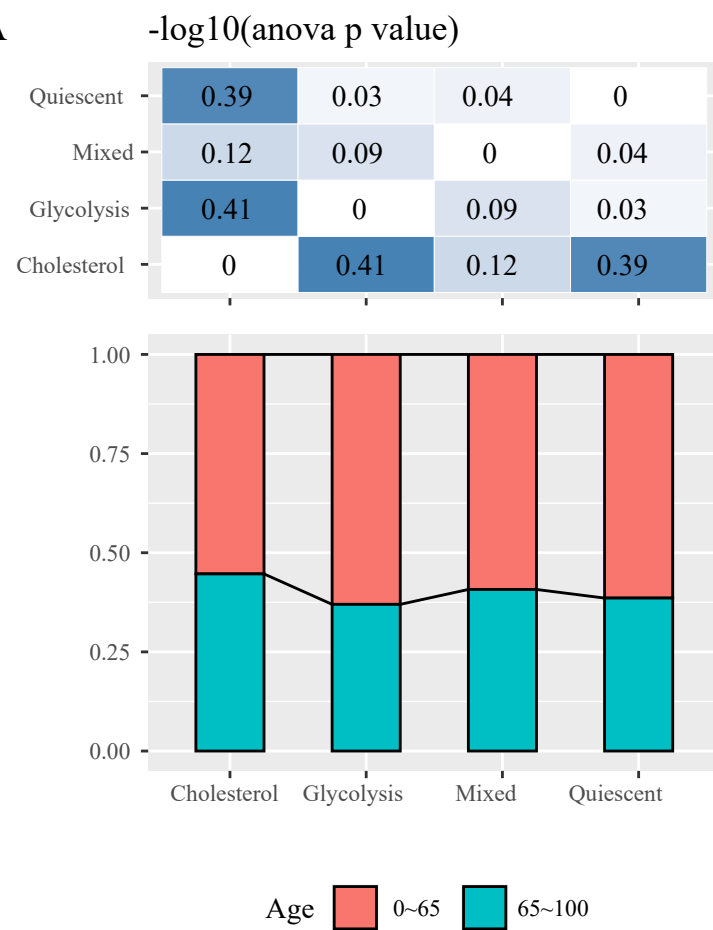

B

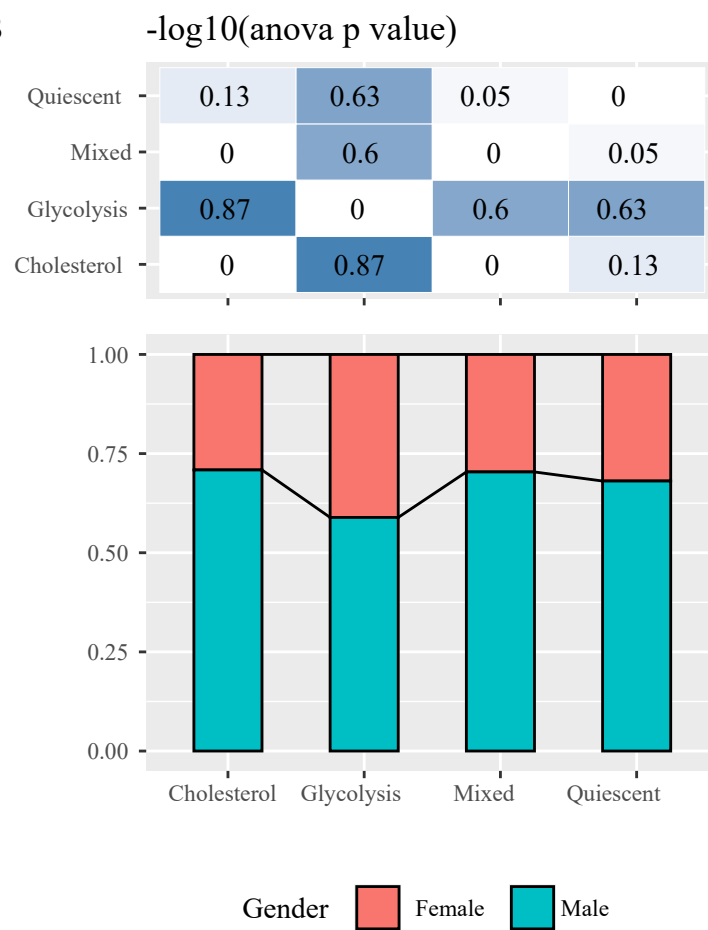

C

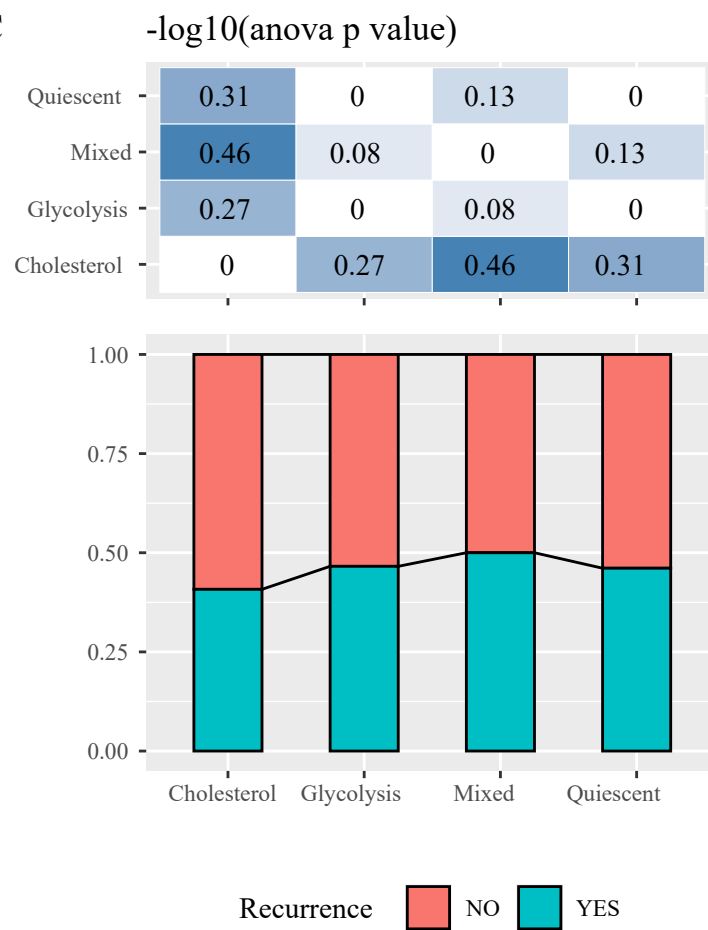

D

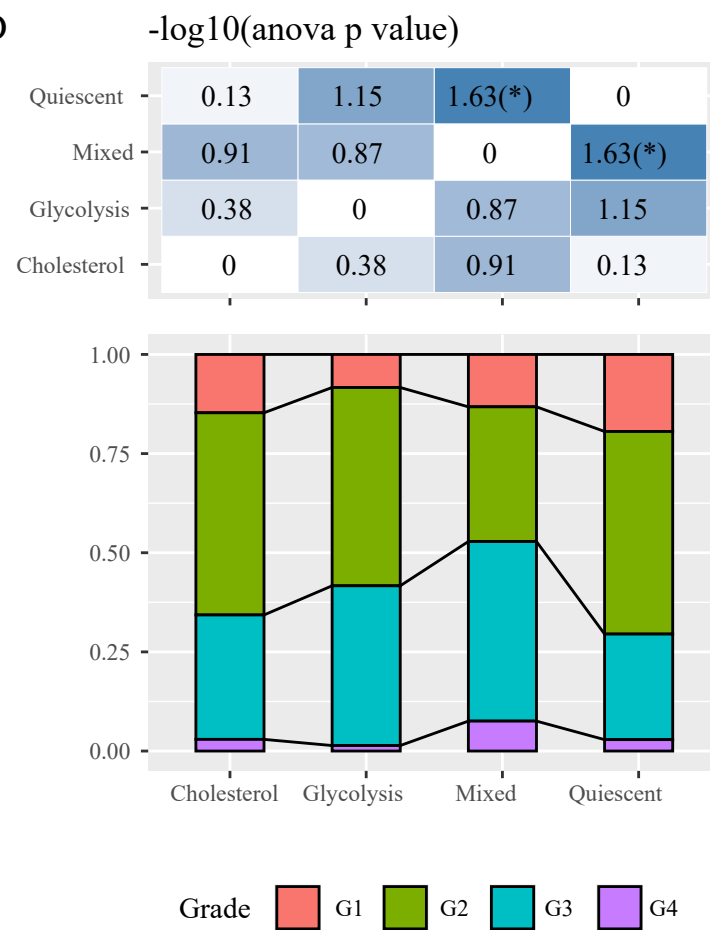

E

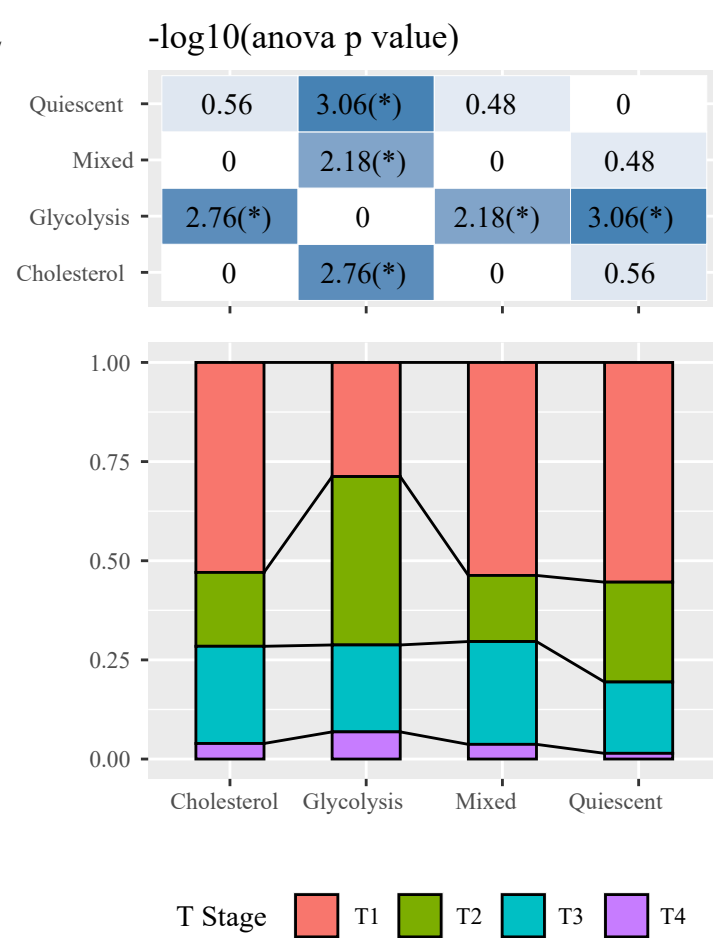

F

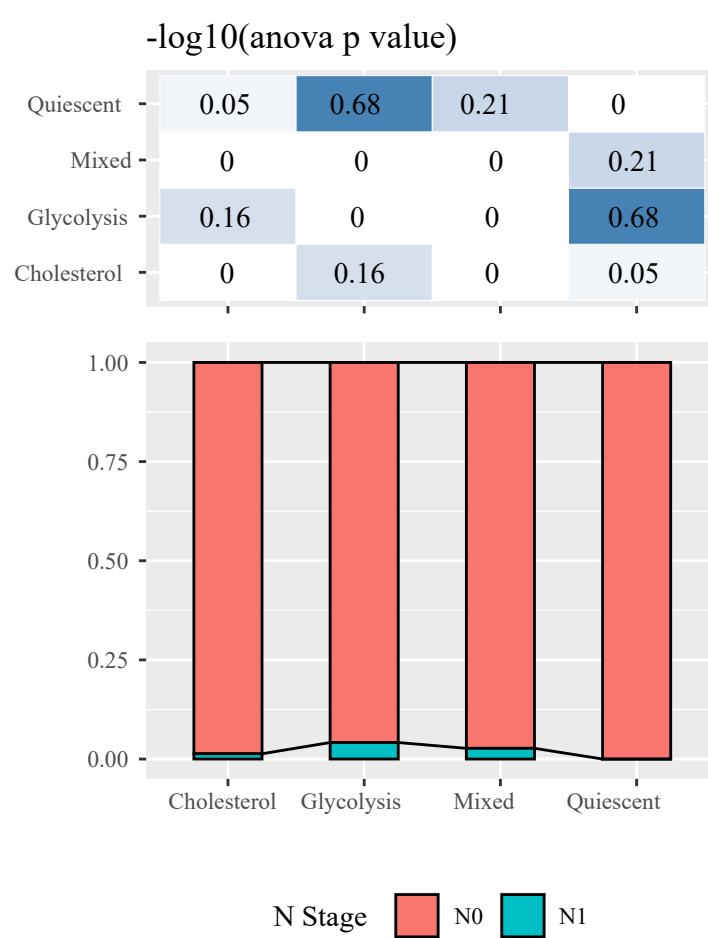

G

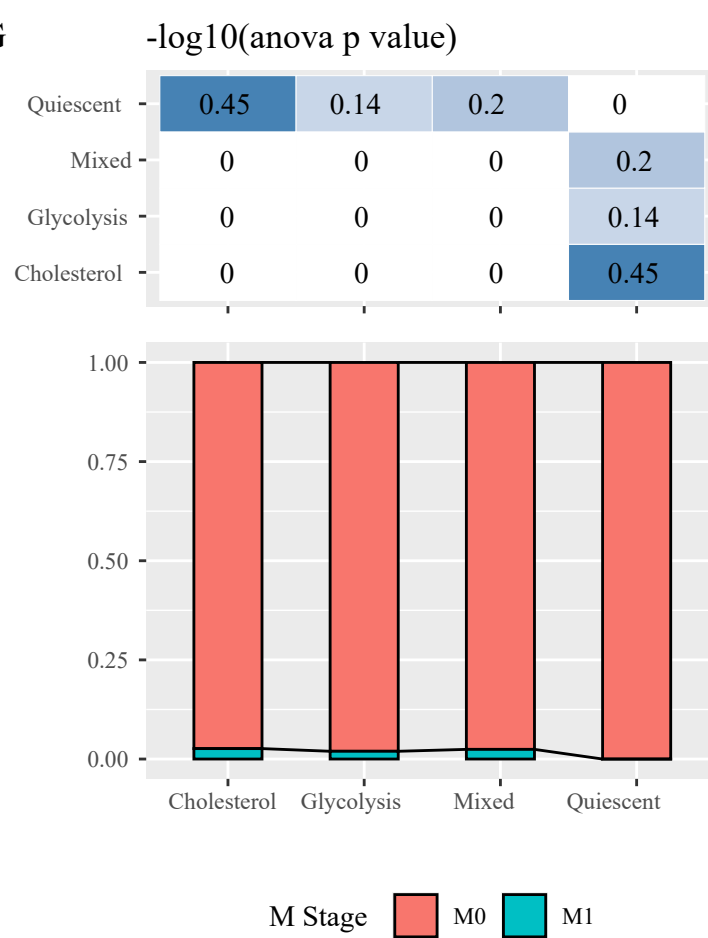

H

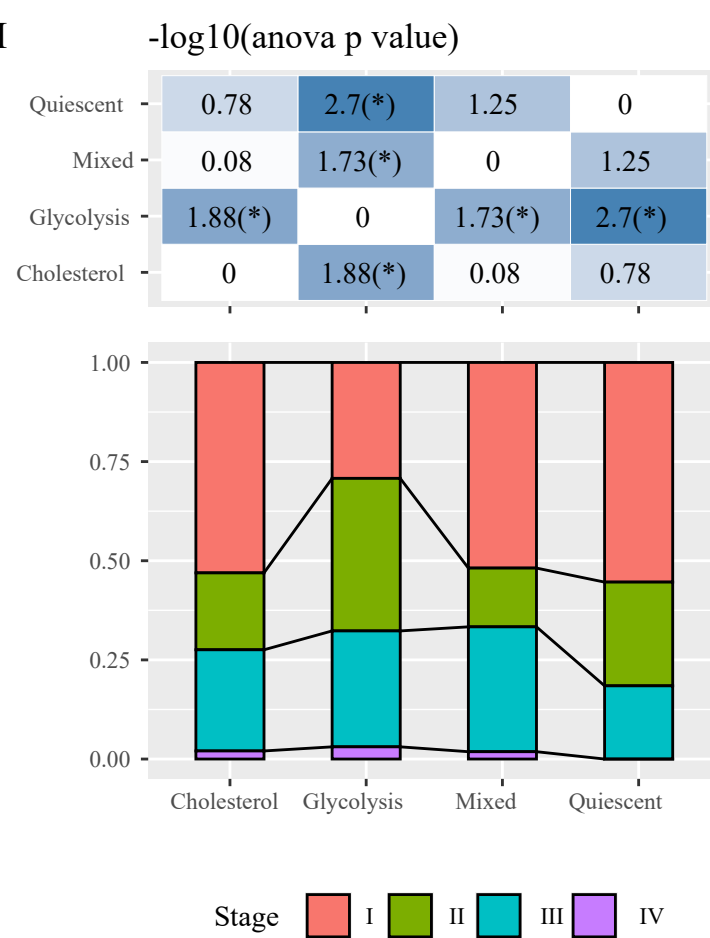

I

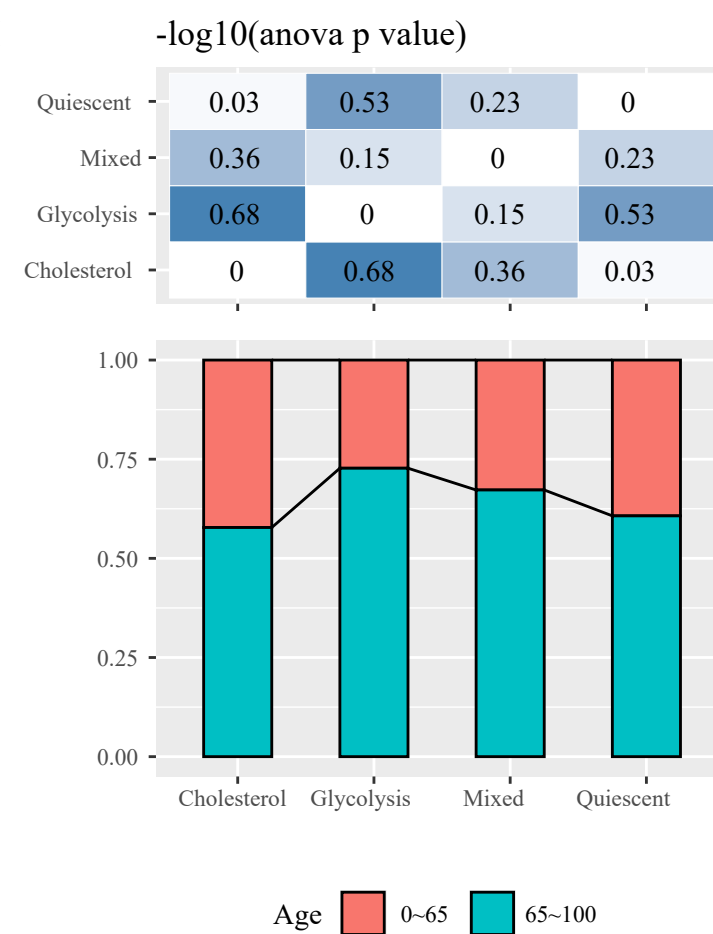

J

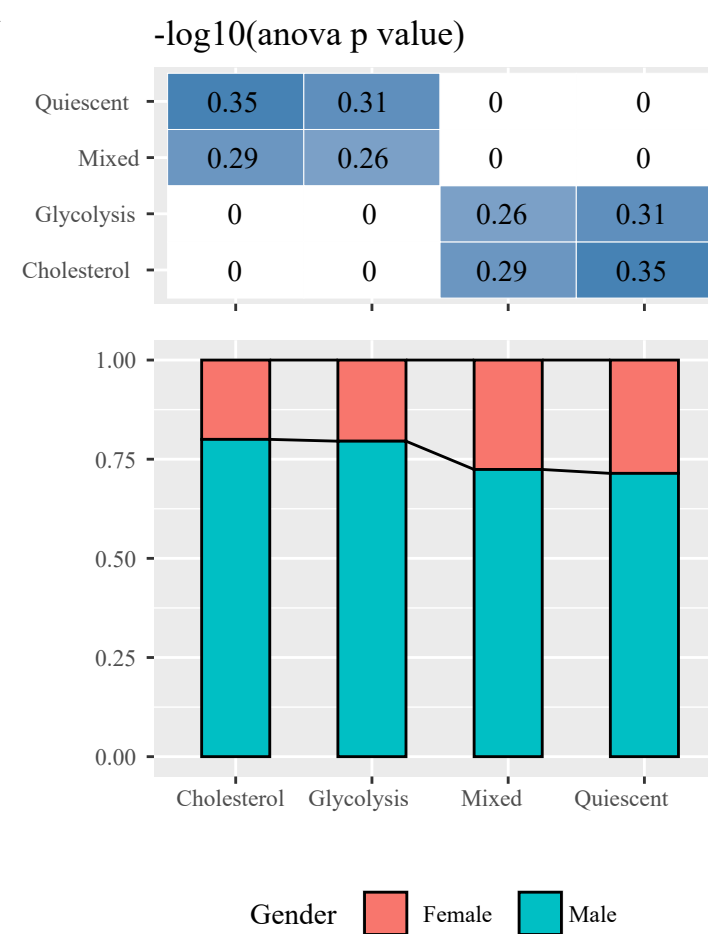

K

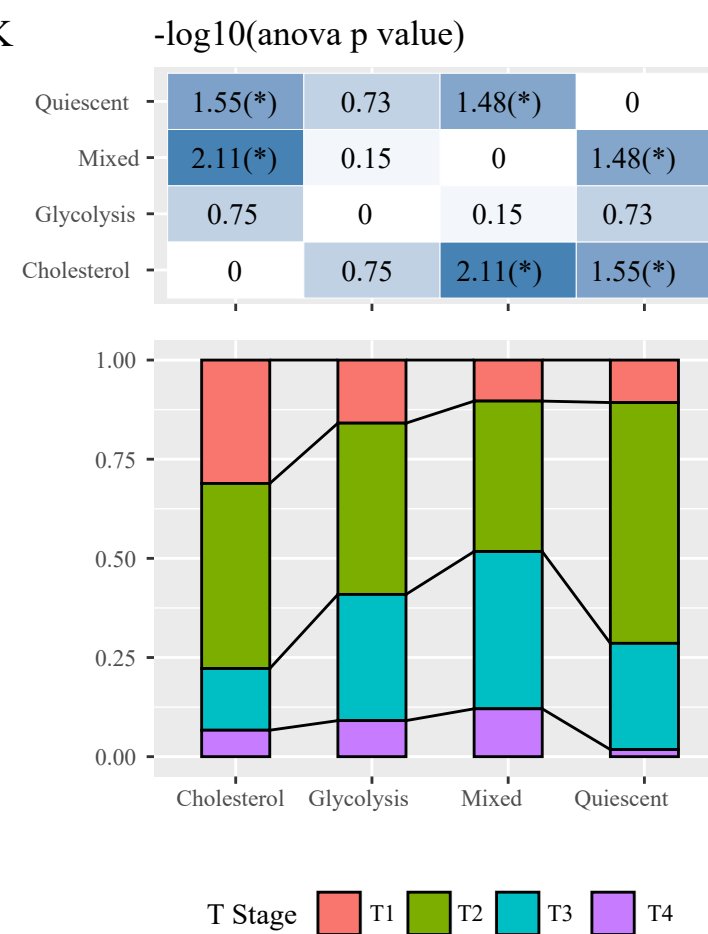

Supplement: Supplementary Materials — Supplementary Figure S1. Comparison between molecular subtype and existing immune molecular subtypes in TCGA dataset. A: Comparison between molecular subtype and existing immune subtypes. B: Distribution comparison of immune subtypes among different molecular subtypes. Supplementary Figure S2. GO functional annotation of MPC1/2 related genes in TCGA dataset. ABC: GO function annotation of genes positively related to MPC1/2. DE: GO function annotation of genes negatively related to MPC1/2. Supplementary Figure S3. Identification and analysis of differentially expressed genes. A: Volcano map of DEGs between Mixed and Quiescent groups in TCGA dataset. B: Heatmap of differentially grouped genes in TCGA dataset mixed and quiet. C: Volcano map of DEGs in Mixed and Quiescent groups of HCCDB18 dataset. D: Heatmap of differentially grouped genes in the HCCDB18 dataset of Mixed and Quiescent groups. Supplementary Figure S4. (A-H) Distribution comparison of different clinical characteristics among four molecular subtypes in TCGA dataset. (I–K) Distribution comparison of different clinical features among four molecular subtypes in the HCCDB18 dataset. Supplementary Figure S5. The estimated distribution of 22 immune cells in four molecular subtypes in TCGA dataset. ns, no significance. ∗P < 0.05, ∗∗P < 0.01, ∗∗∗P < 0.001, ∗∗∗∗P < 0.0001. Supplementary Table S1. A list of 44 glycolysis/cholesterol-related genes. [file 2014625.f1.zip › Supplementary Figure S3.pdf]

A

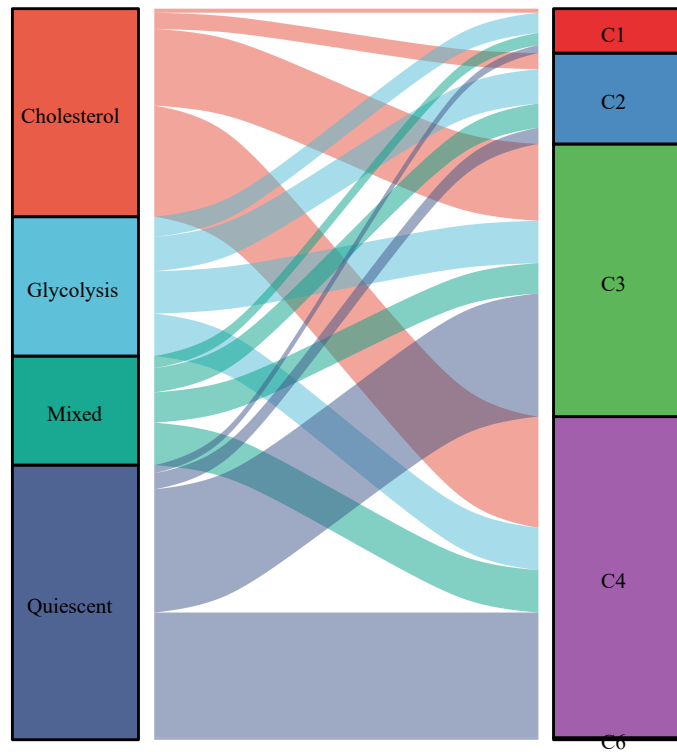

B

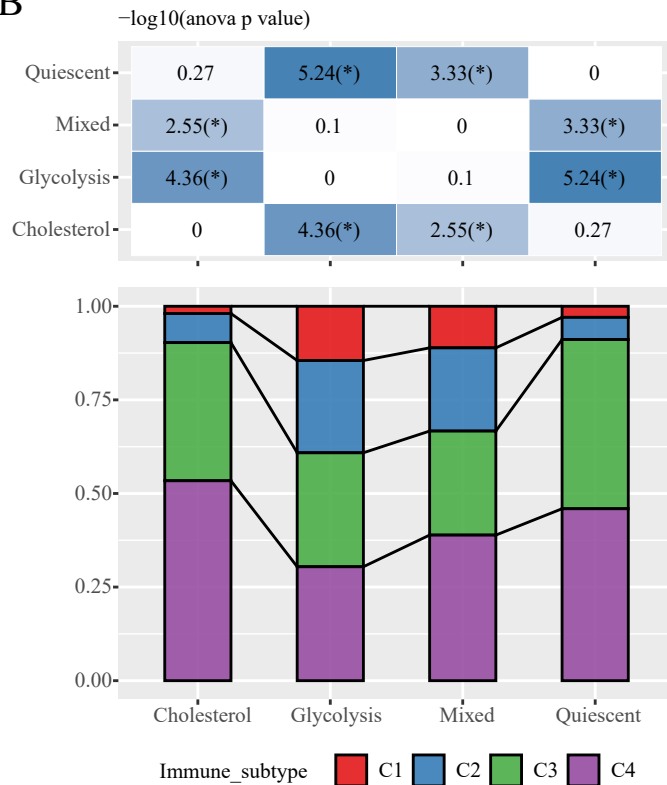

Supplement: Supplementary Materials — Supplementary Figure S1. Comparison between molecular subtype and existing immune molecular subtypes in TCGA dataset. A: Comparison between molecular subtype and existing immune subtypes. B: Distribution comparison of immune subtypes among different molecular subtypes. Supplementary Figure S2. GO functional annotation of MPC1/2 related genes in TCGA dataset. ABC: GO function annotation of genes positively related to MPC1/2. DE: GO function annotation of genes negatively related to MPC1/2. Supplementary Figure S3. Identification and analysis of differentially expressed genes. A: Volcano map of DEGs between Mixed and Quiescent groups in TCGA dataset. B: Heatmap of differentially grouped genes in TCGA dataset mixed and quiet. C: Volcano map of DEGs in Mixed and Quiescent groups of HCCDB18 dataset. D: Heatmap of differentially grouped genes in the HCCDB18 dataset of Mixed and Quiescent groups. Supplementary Figure S4. (A-H) Distribution comparison of different clinical characteristics among four molecular subtypes in TCGA dataset. (I–K) Distribution comparison of different clinical features among four molecular subtypes in the HCCDB18 dataset. Supplementary Figure S5. The estimated distribution of 22 immune cells in four molecular subtypes in TCGA dataset. ns, no significance. ∗P < 0.05, ∗∗P < 0.01, ∗∗∗P < 0.001, ∗∗∗∗P < 0.0001. Supplementary Table S1. A list of 44 glycolysis/cholesterol-related genes. [file 2014625.f1.zip › Supplementary Figure S4 (1).pdf]

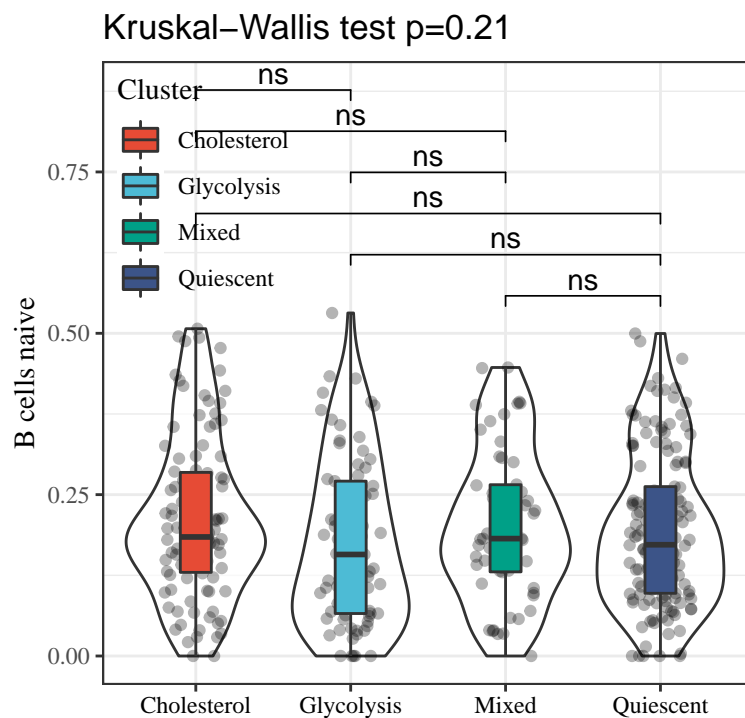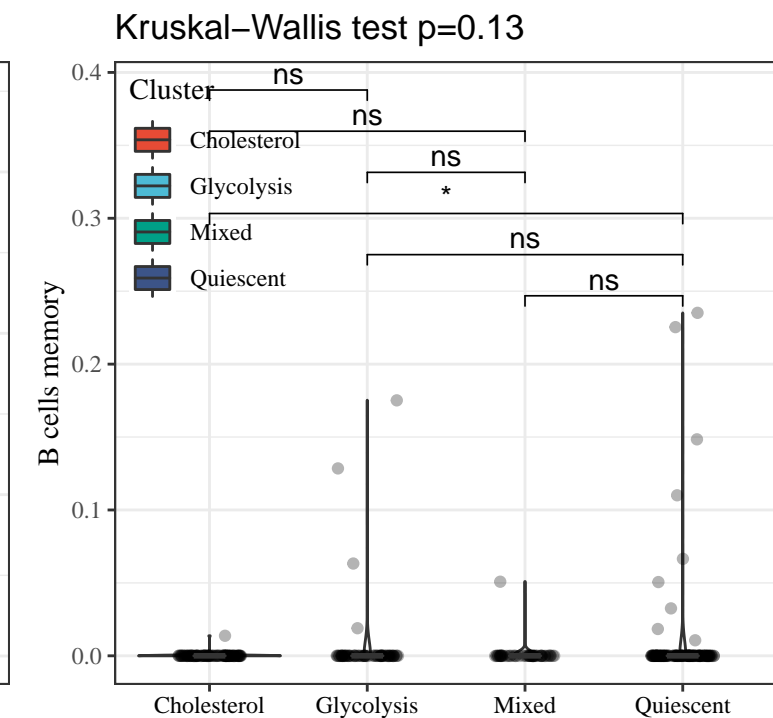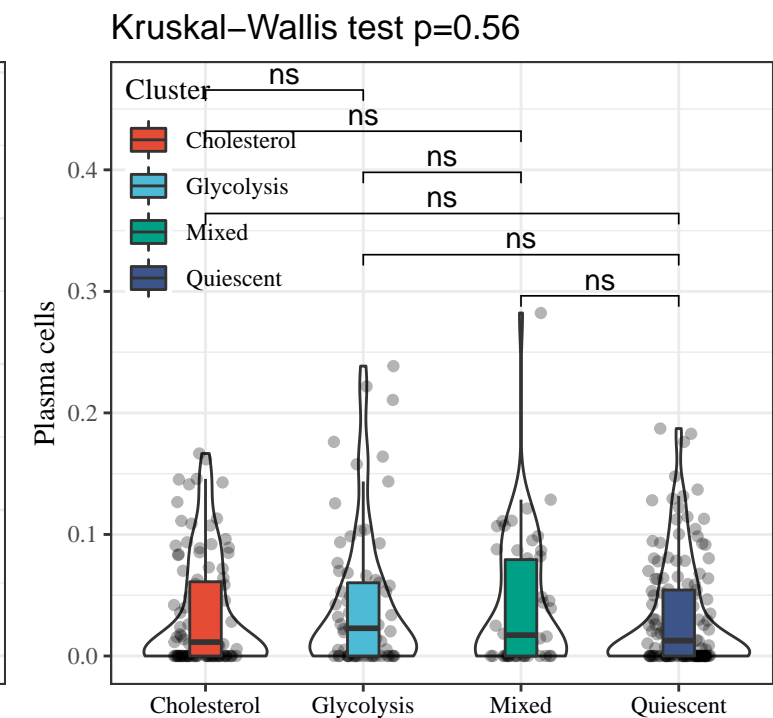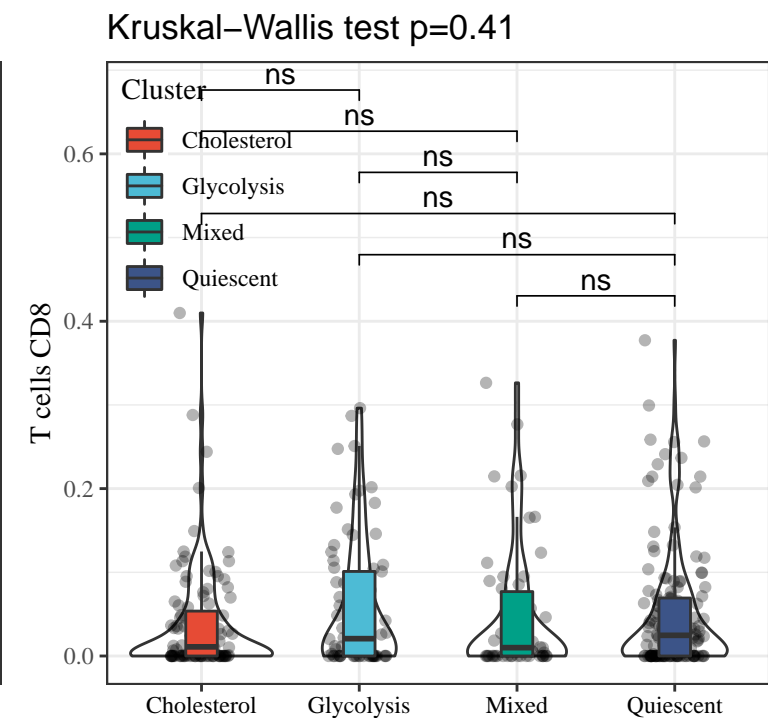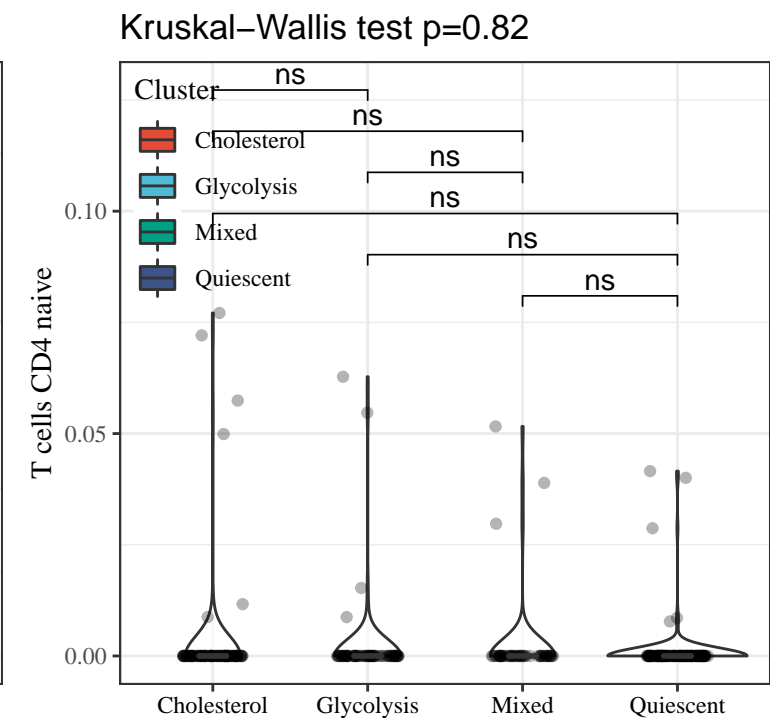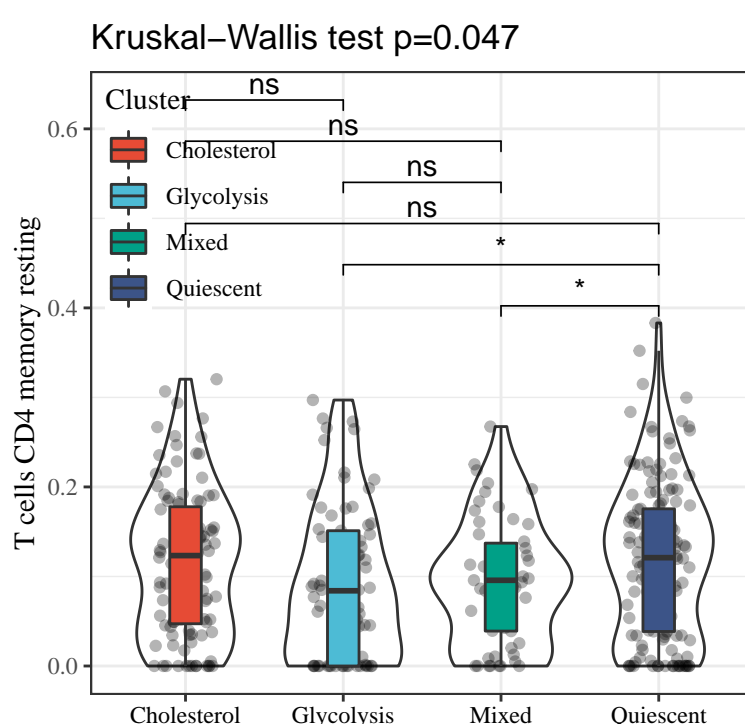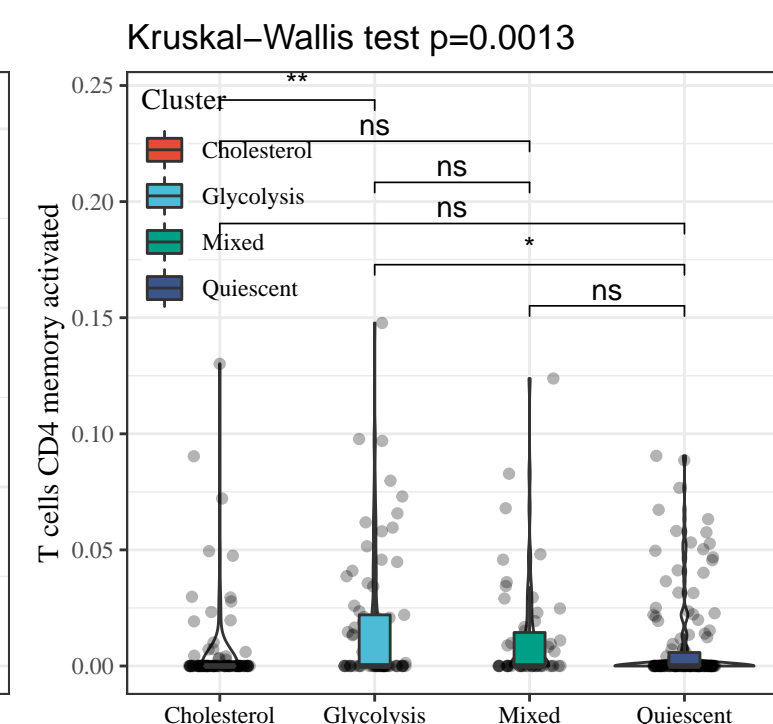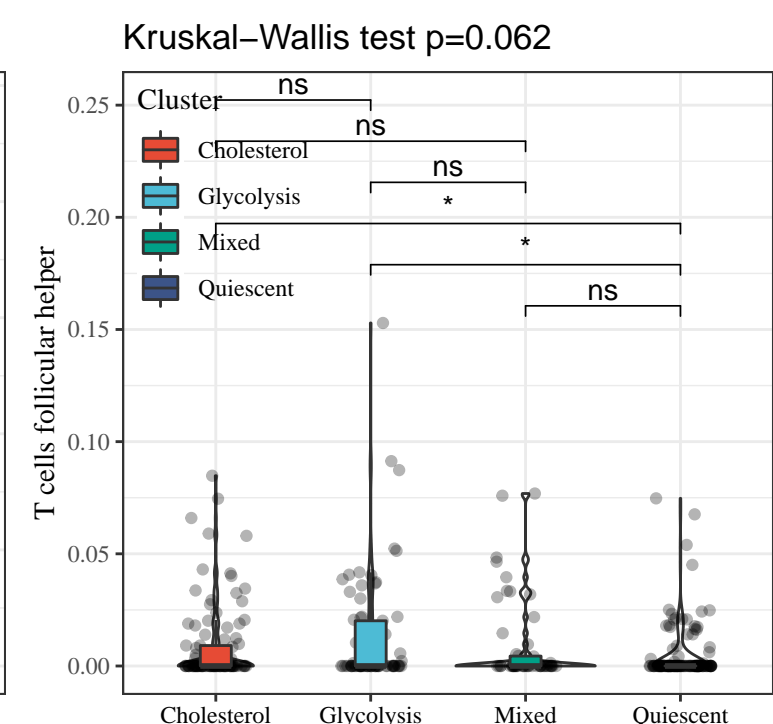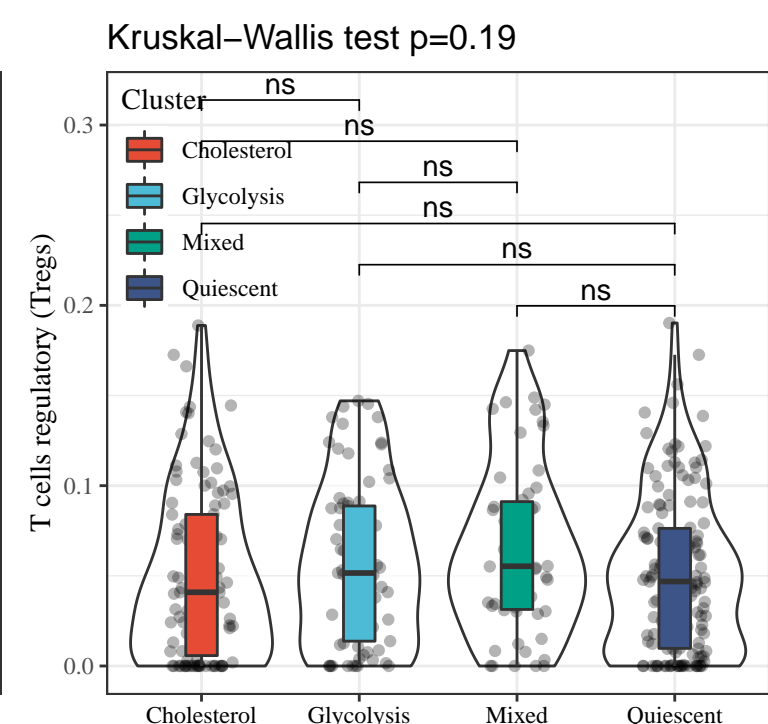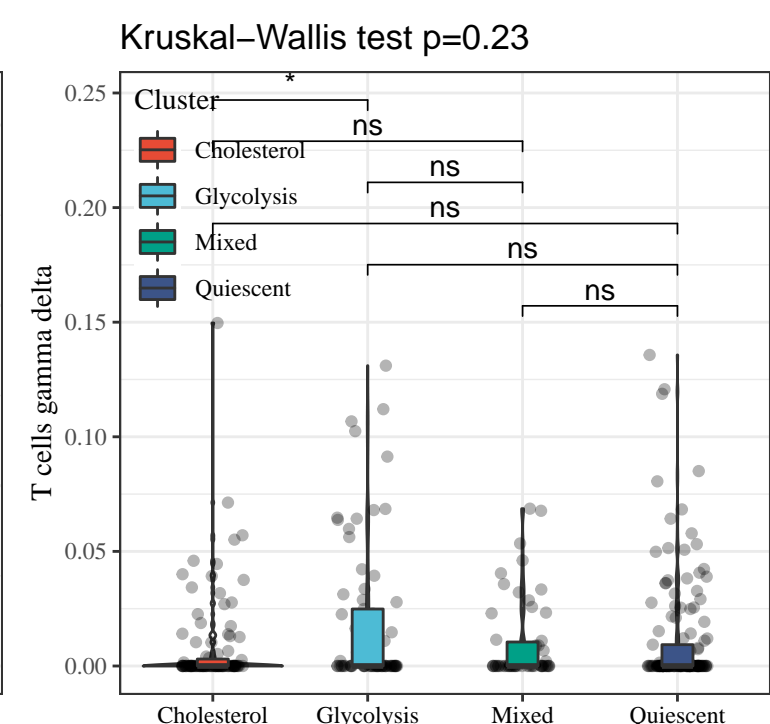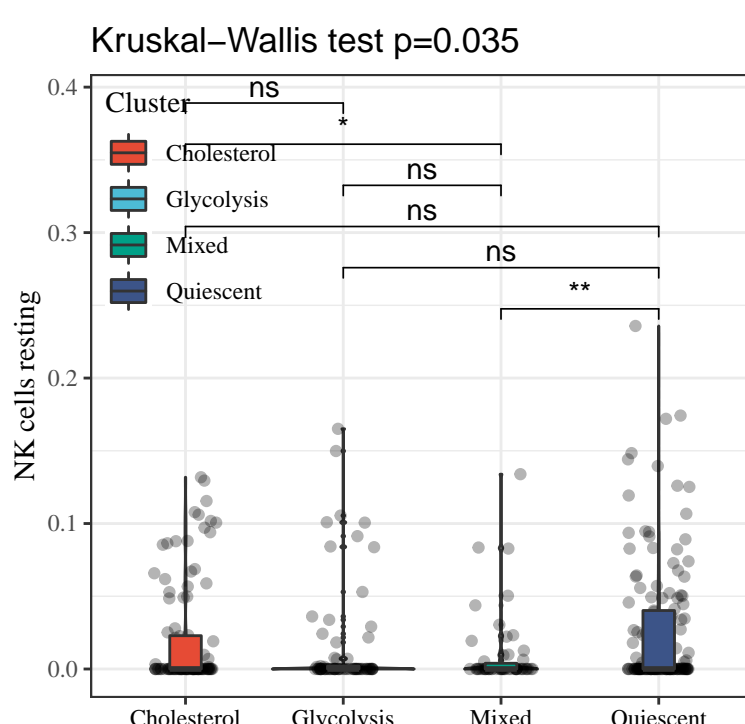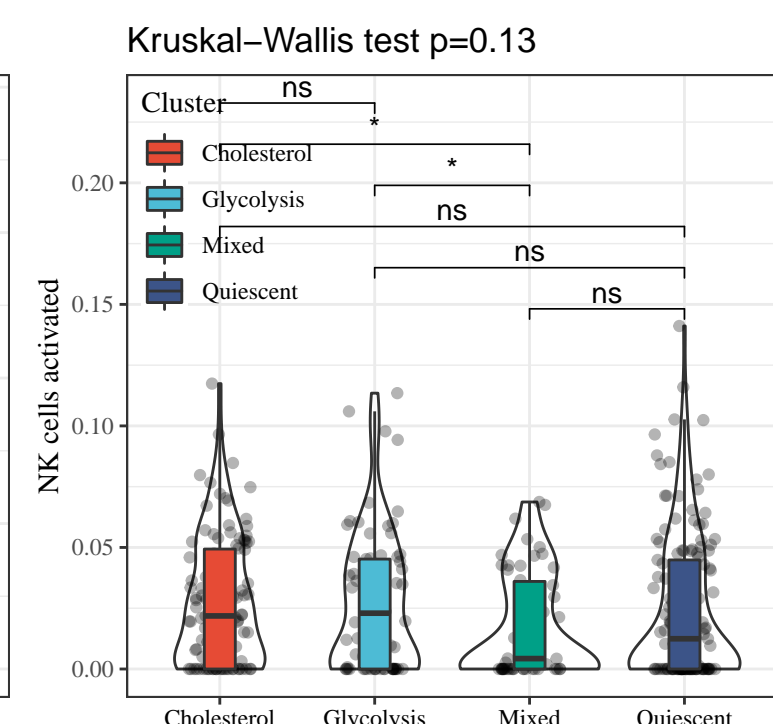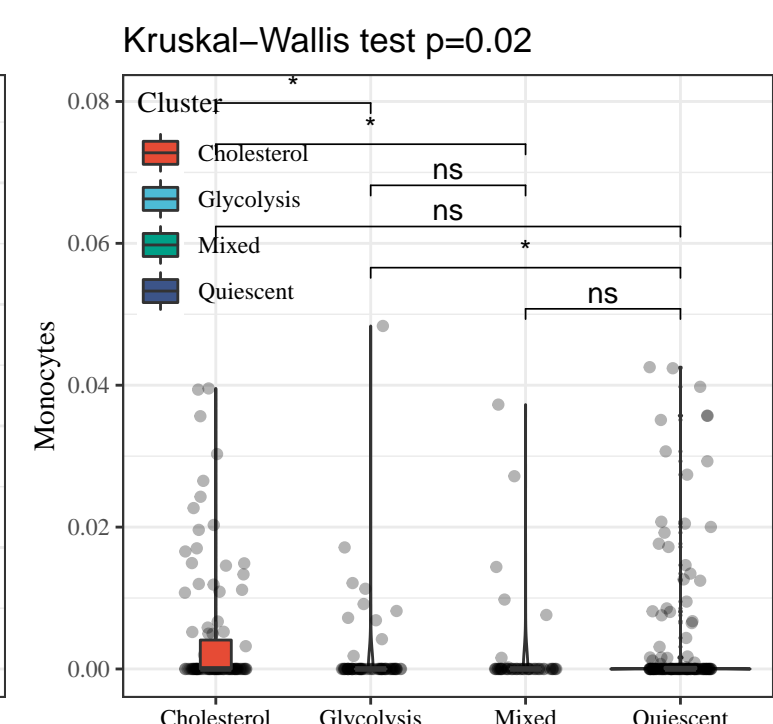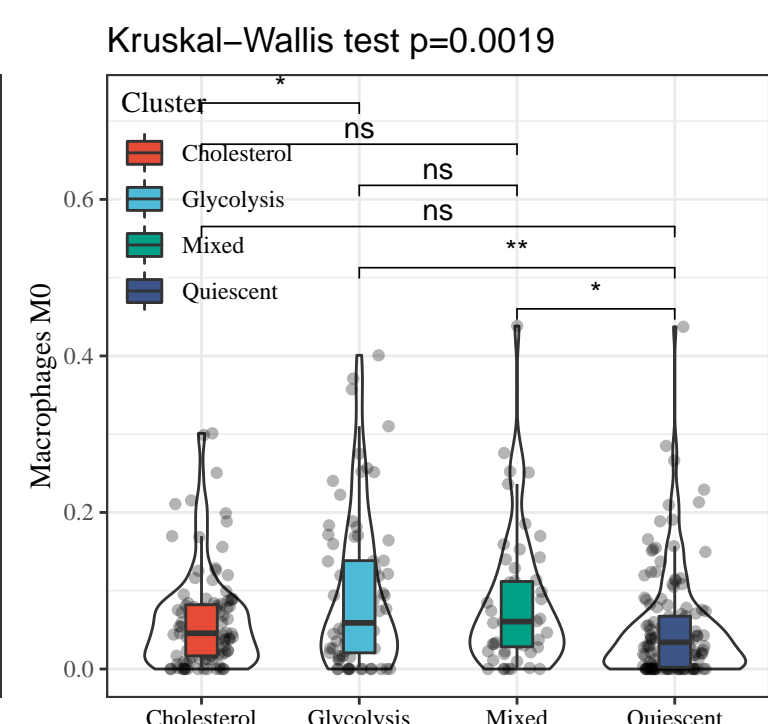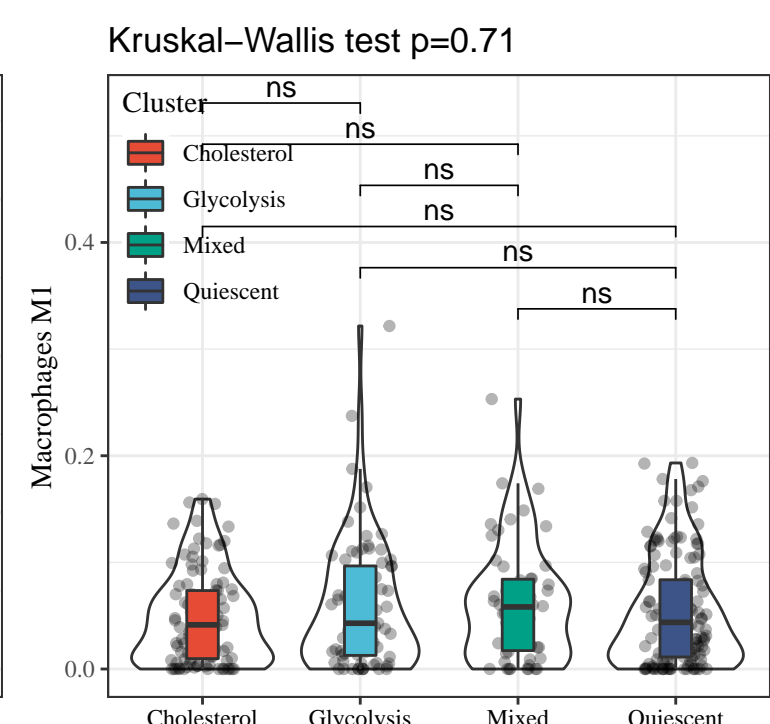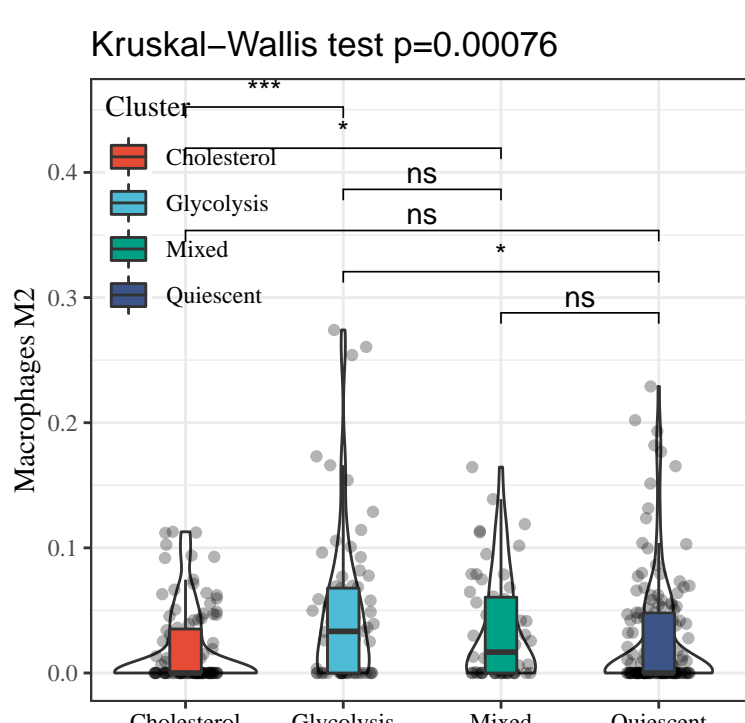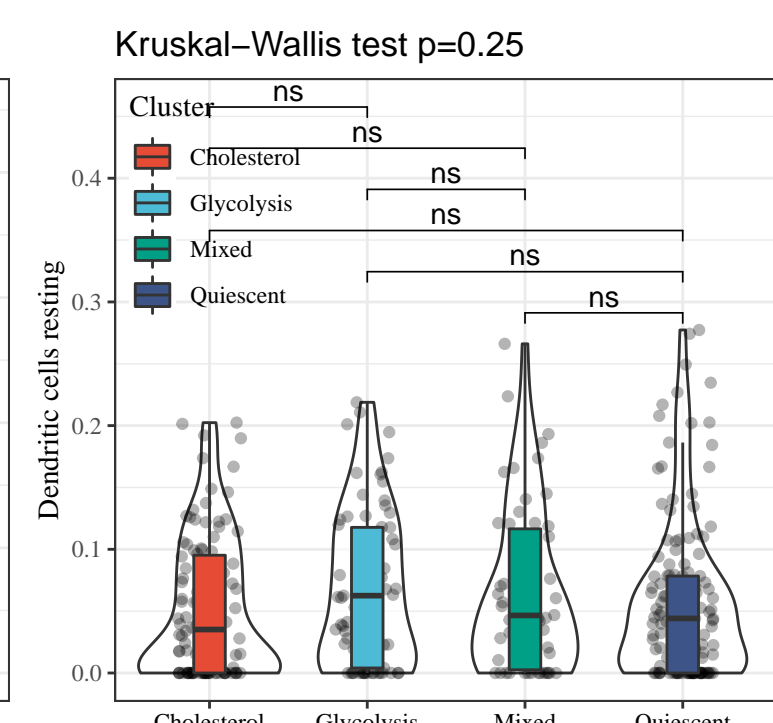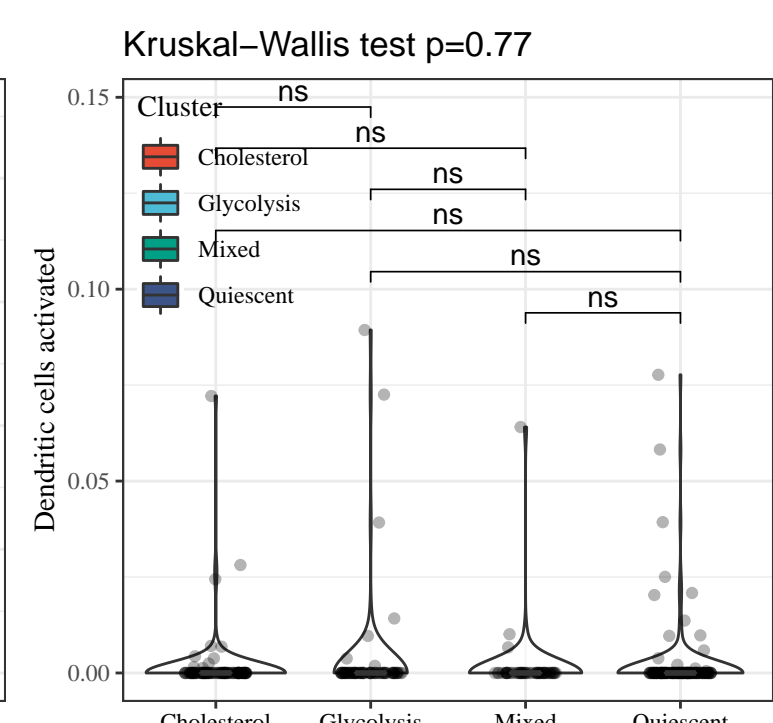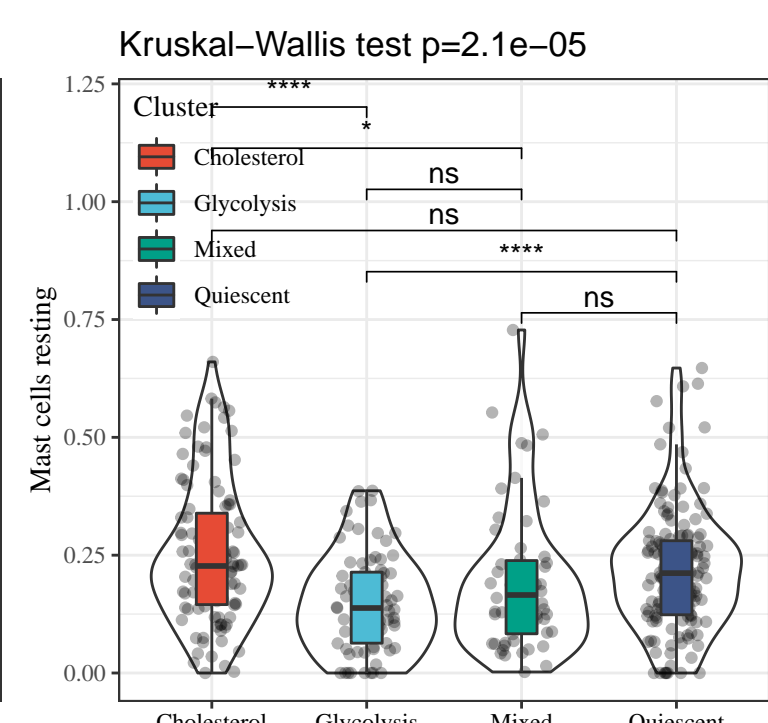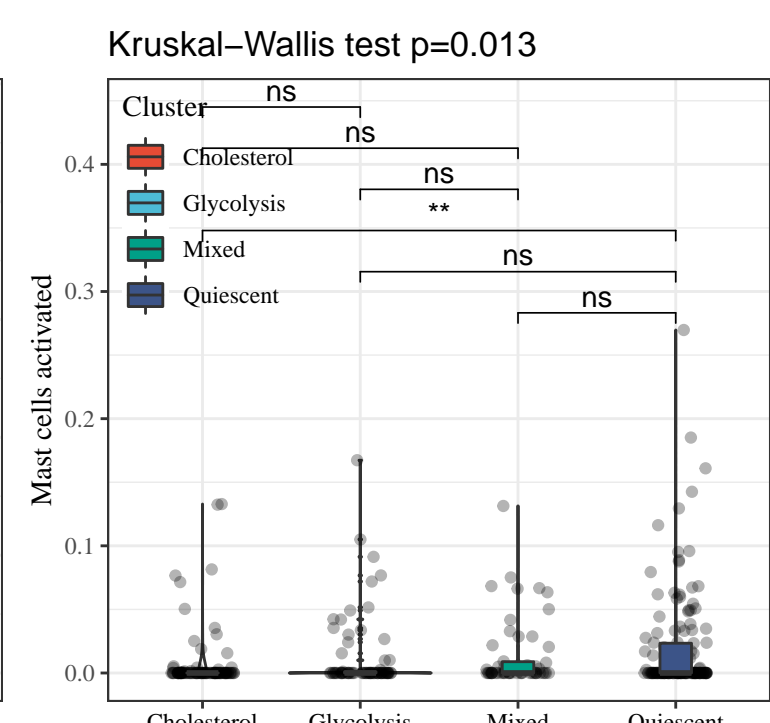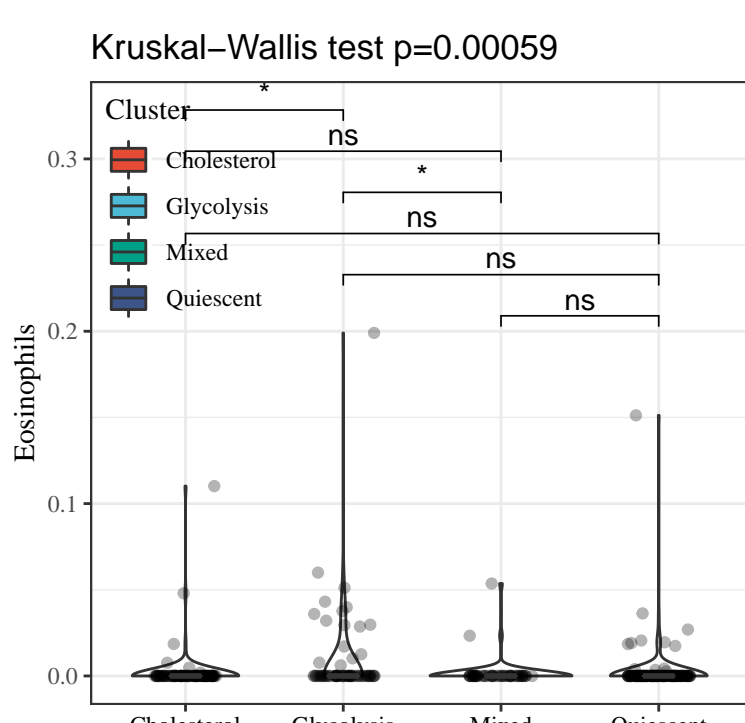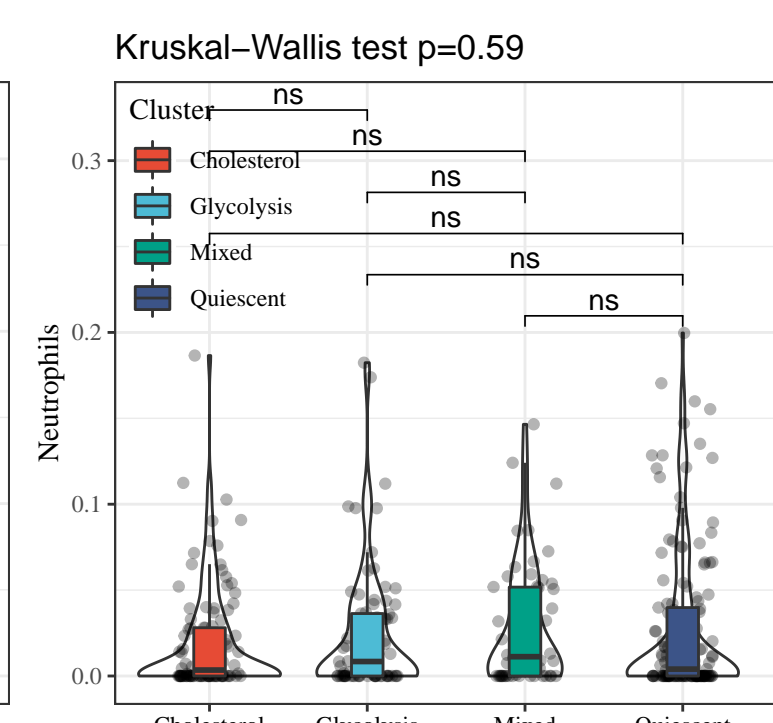

Supplement: Supplementary Materials — Supplementary Figure S1. Comparison between molecular subtype and existing immune molecular subtypes in TCGA dataset. A: Comparison between molecular subtype and existing immune subtypes. B: Distribution comparison of immune subtypes among different molecular subtypes. Supplementary Figure S2. GO functional annotation of MPC1/2 related genes in TCGA dataset. ABC: GO function annotation of genes positively related to MPC1/2. DE: GO function annotation of genes negatively related to MPC1/2. Supplementary Figure S3. Identification and analysis of differentially expressed genes. A: Volcano map of DEGs between Mixed and Quiescent groups in TCGA dataset. B: Heatmap of differentially grouped genes in TCGA dataset mixed and quiet. C: Volcano map of DEGs in Mixed and Quiescent groups of HCCDB18 dataset. D: Heatmap of differentially grouped genes in the HCCDB18 dataset of Mixed and Quiescent groups. Supplementary Figure S4. (A-H) Distribution comparison of different clinical characteristics among four molecular subtypes in TCGA dataset. (I–K) Distribution comparison of different clinical features among four molecular subtypes in the HCCDB18 dataset. Supplementary Figure S5. The estimated distribution of 22 immune cells in four molecular subtypes in TCGA dataset. ns, no significance. ∗P < 0.05, ∗∗P < 0.01, ∗∗∗P < 0.001, ∗∗∗∗P < 0.0001. Supplementary Table S1. A list of 44 glycolysis/cholesterol-related genes. [file 2014625.f1.zip › Supplementary Figure S5.pdf]
